# Platelet-Rich Fibrin Membrane as a Personalized Autologous Graft Layer for Hypospadias Surgery: Systematic Review and Meta-Analysis

**DOI:** 10.3390/jpm16100527

**Published:** 2026-10-12

**Authors:** George Pantalos, Smaragda Papachristidou, Konstantinos Velaoras, Christos Plataras, Ioannis Alexandrou, Nikolaos Zavras, George Vaos

**Affiliations:** 1 Department of Paediatric Surgery, Penteli General Children’s Hospital, 15236 Athens, Greece; kvelaoras@yahoo.gr (K.V.); christosplataras@yahoo.gr (C.P.); inalexandrou@hotmail.com (I.A.); 2 Paediatric Emergency Department, P.&A. Kyriakou Children’s Hospital, 11527 Athens, Greece; smagia.gsp@gmail.com; 3 Department of Paediatric Surgery, Medical School, National and Kapodistrian University of Athens, 11527 Athens, Greece; nzavras@med.uoa.gr (N.Z.); gvaos@med.uoa.gr (G.V.)

**Keywords:** hypospadias, platelet-rich fibrin, urethroplasty, urethral fistula, coverage layer

## Abstract

**Background/Objectives**: Hypospadias is a congenital malformation where surgical treatment remains complex while complications are common. Coverage of the neourethra with various layers, such as dartos flaps, is usually part of surgical reconstruction. Coverage layers have been associated with a reduction in complications. Recently platelet-rich fibrin (PRF), a second-generation personalized autologous blood-derived product, has been used as a protective layer after being formed into a membrane. **Methods**: A systematic review and meta-analysis were conducted by searching the PubMed/MEDLINE and Scopus databases for relevant studies to assess differences in postoperative outcomes when using a PRF membrane as a coverage layer for hypospadias urethroplasty. **Results**: Eight studies were eligible. Operative time is slightly extended with PRF application (mean difference = 5.02 min, 95% CI: 2.76 to 7.29, *p* < 0.0001), while hospital stay appears to be equivalent. Urethral fistula (Risk Ratio (RR) = 0.55, 95% CI: 0.35 to 0.88, *p* = 0.01), meatal stenosis (RR = 0.41, 95% CI: 0.17 to 1.00, *p* = 0.05), wound infection (RR = 0.42, 95% CI: 0.17 to 1.04, *p* = 0.06) and bleeding rates (RR = 0.55, 95% CI: 0.12 to 2.46, *p* = 0.43) present favorable results when using a PRF layer. Urethral stricture risk cannot be estimated. Glans dehiscence rates appear higher when PRF membranes are used. However, heterogeneity of the literature was high and certainty of evidence was low. **Conclusions**: PRF layer application seems promising in reducing postoperative complication rates of hypospadias surgery, except for glans dehiscence. Standardization of PRF production, application and long-term follow-up of patients are important for future studies and strengthening the body of evidence.

## 1. Introduction

Hypospadias is among the most common male congenital anomalies, characterized by malposition of the urethral meatus on the ventral surface of the penis. It can be associated with other concurrent congenital anomalies or manifest as a solitary deformity [1,2]. The severity of hypospadias can be variable and is dependent on the location of the urethral meatus, urethral plate characteristics, associated chordee and other factors [3]. In general, distal location of the meatus lies on the less severe side of the hypospadias malformation spectrum. In contrast, proximal hypospadias can be regarded as a more severe and complex malformation to reconstruct [4]. Hypospadias severity translates directly to an increased percentage of complications for complex cases [5]. Urethral plate and malformed adjacent tissue vascularity and local healing impairment may contribute to the aforementioned fact, along with the more complex surgical techniques used.

Surgical reconstruction of hypospadias aims to improve both functional and cosmetic outcomes for hypospadias. Many techniques have been employed historically with variable outcomes [6,7]. Staged procedures are typically employed for more severe cases, commonly mid-penile and proximal cases [8]. These procedures can have two or three stages and include chordee correction and penile straightening, grafting of tissue if local tissue is inadequate and urethroplasty. Some techniques are modifications of popular ones, with the most widely used recent example being the Tubularized Incised Plate (TIP) urethroplasty, a modification of the Thiersch-Duplay procedure [9,10]. Other techniques employ local flaps for repair, like the Mathieu flip-flap procedure or Duckett’s procedure [11,12,13]. Similarly, procedures such as Bracka or Snodgraft repair typically utilize grafts from preputial skin or buccal mucosa [14,15,16]. It is important to note that hypospadias surgeons should familiarize themselves with all relevant techniques, as every hypospadias surgeon must have a strategy and technical armamentarium to provide personalized care [17]. It is imperative that the surgeon modify the reconstruction strategy intraoperatively to provide personalized surgery suited for each child’s unique problem.

Complications of surgery for hypospadias include urethral or urethrocutaneous fistula, wound or glans dehiscence, bleeding or hematoma needing surgery, wound infection, meatal stenosis, urethral stricture, urethral diverticula and others [5,18,19,20]. Hypospadias patients often need multiple operations, either as part of staged reconstruction or as part of complication repair, commonly urethral fistula closure [21]. Mention should be given to so-called hypospadiac cripples, patients having undergone multiple operations that still need salvage operations [17,22].

The most common complication of hypospadias is urethral fistula, usually needing operative correction [23,24]. A popular and usual technique used to reduce rates of urethral fistula and wound dehiscence is to apply a coverage layer over the urethroplasty [25]. This improves vascularity and thus local healing while also providing a tissue mechanical barrier to support and buttress the urethroplasty. Avoiding overlapping suture lines is a technical note to reduce urethral fistula rates [23,26,27]. A multitude of options for this protective coverage layer are available, including local dartos flaps, dorsal penile dartos transposition flaps, tunica vaginalis flaps, fibrin glue and others [25,28,29]. Fibrin glue has been used to cover the urethroplasty, with theoretical advantages being the easy application and wound healing promotion [30,31,32].

One of the most recent additions as an option for the protective layer of urethroplasty coverage is the platelet-rich fibrin (PRF) membrane. PRF is a second-generation platelet-rich product derived from the patient’s blood [33]. This provides a personalized autologous membranous graft to cover the urethroplasty as a barrier and, in tandem, provides healing factors to support the healing process and minimize infection. PRF contains platelets, leukocytes, growth factors and it can be produced during surgery; it can be fashioned into a thin membrane, ideal for application as a layer. Application of PRF has been widespread in multiple medical fields, including pediatric, oral, plastic and general surgery, with favorable outcomes. However, special consideration should be given to the variability of PRF formulations, naming and relevant conventions, since authors could be using PRF and using different naming schemes or vice versa [34].

This systematic review and meta-analysis aims to gather all relevant clinical studies in the field of PRF application as a urethroplasty coverage layer during hypospadias surgery and document its effects on patient and surgical outcomes. Recent clinical studies have used PRF during primary or re-do cases. Application of PRF membrane has been described as a solitary or as a second layer over another, such as a dartos flap. Although past reviews have studied the subject, this systematic review and meta-analysis aims to provide a complete overview of the field to inform interested readers on the feasibility of the use of PRF for hypospadias operations. For this reason, the included studies describe the use of a membranous PRF layer over the urethroplasty, even if it is named otherwise, while excluding all studies not involving application of a PRF membrane as a graft layer. It is interesting to monitor this field for the true need and usefulness of PRF membrane in hypospadias surgery and whether it is one more weapon in the surgeon’s armamentarium or a layer that should be broadly used, regardless of patient and hypospadias characteristics or technique selection.

## 2. Materials and Methods

### 2.1. Study Protocol

**Figure 1:**
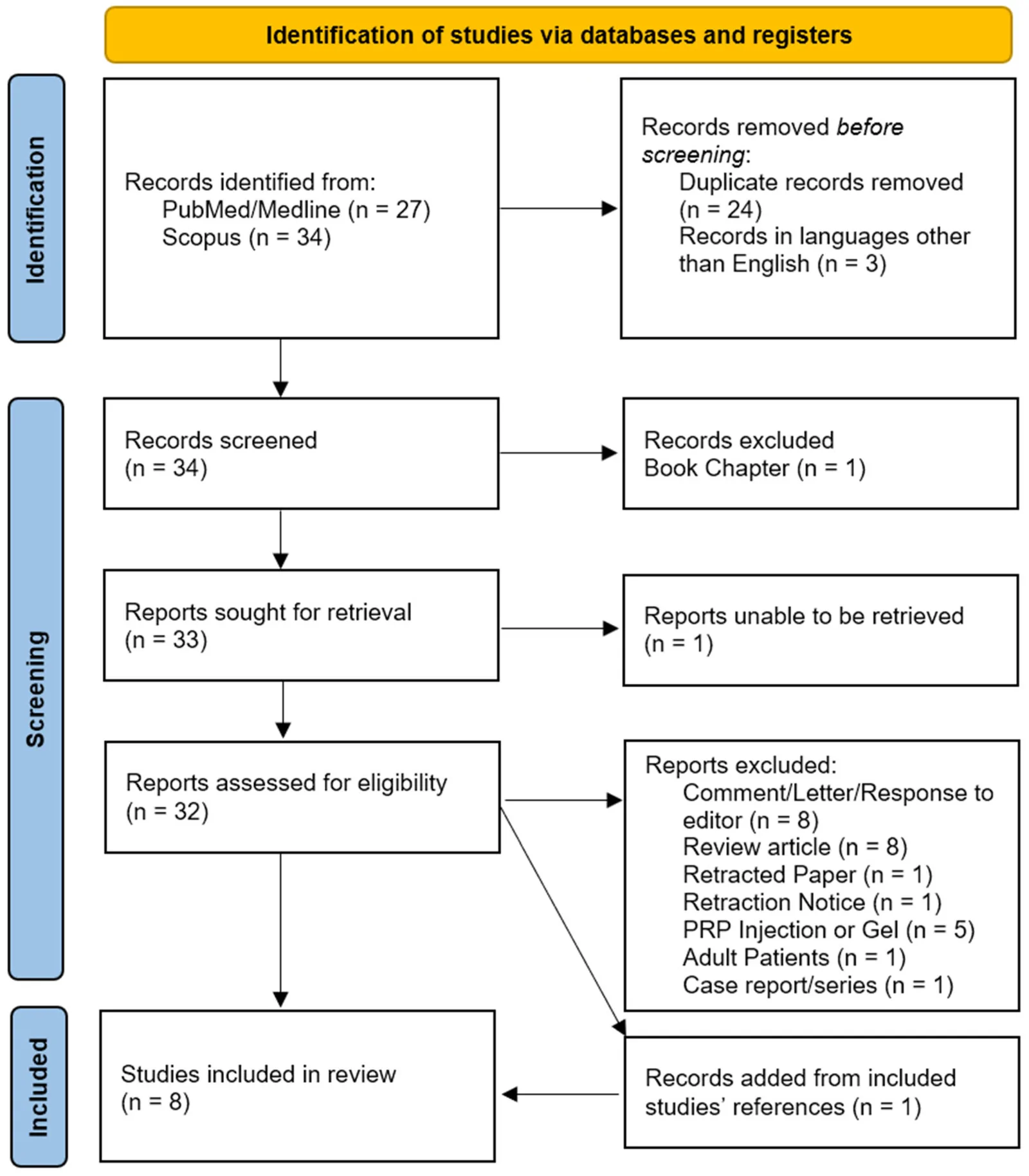
PRISMA flow diagram for the records identified after database searching.

This systematic review and meta-analysis is reported according to the Preferred Reporting Items for systematic review and meta-analysis (PRISMA) guidelines (Figure 1) [35]. This review was not registered and a protocol was not formally prepared. Consequently, there were no amendments to the protocol information. The completed PRISMA 2020 Checklist has been provided in Supplementary Table S1.

### 2.2. Search Strategy

A systematic literature search was completed to identify studies related to the use of PRF during surgical treatment of hypospadias. Articles including clinical data on the application of a PRF membrane graft as a protective layer during hypospadias repair were screened. The description of the production process of the personalized autologous graft was evaluated, as well as the surgical technique used for primary or re-operative hypospadias repair. The influence of PRF graft usage with regard to the outcomes and the potential avoidance of complications was also examined.

We searched two major databases (PubMed/MEDLINE and Scopus) until 15 September 2026. Searches were conducted using the keywords “platelet”, “rich”, “fibrin”, “plasma” and “hypospadias”. Specific search terms used are provided in Appendix A. Only randomized controlled trials (RCTs) and comparative clinical studies (CCTs) written in English were included, while editorials, comments, letters or responses to the editor and review articles were excluded. Retracted papers and associated retraction notices were also excluded. After deduplication, abstracts underwent screening for relevance to this research topic during the initial search. Following retrieval of the aforementioned articles, a full-text evaluation was done to assess each study for inclusion. References from the studies included were also screened to ensure a thorough literature review.

### 2.3. Eligibility Criteria

Selection criteria included original clinical studies of prospective or retrospective design, i.e., RCTs or CCTs with results on the application of PRF as an autologous graft reinforcement layer for surgical repair of pediatric hypospadias. Selected studies had to compare surgical repair of hypospadias using PRF as a sole or adjunctive urethroplasty coverage layer versus repair without PRF. No restrictions were imposed as to the location of hypospadias, technique or other characteristics. Eligible studies should report clinical outcomes including operative time, length of hospital stay and complications including urethral fistula or strictures, meatal stenosis, wound infections, bleeding or glans dehiscence.

We excluded studies pertaining to the use of Platelet-Rich Plasma (PRP) in injectable or gel forms and not graft-type forms such as membranes. Adult-related literature was also excluded. Editorials or letters on published articles, case reports, retracted papers and non-pediatric studies were also excluded. Similarly, non-available full-text articles and articles in languages other than English were also excluded. Systematic or other reviews were excluded.

Eligible studies should describe PRF production and formation into a membrane with no additives and a single centrifugation cycle. Further information on PRF membrane composition has been provided in Section 3.4 of this review.

### 2.4. Study Screening

Two authors (G.P. and S.P.) independently screened the titles and abstracts of the articles for inclusion. A senior author (G.V.) verified the accuracy of the collection process and resolved any disagreements. Full-text articles were thoroughly read and evaluated by two authors (G.P. and S.P.) and after the removal of duplicated and excluded articles, a final review was conducted by four authors (G.P., S.P., N.Z. and G.V.) independently and disputes were resolved by consensus.

### 2.5. Data Extraction

All authors, after thorough study of literature, determined the data to be extracted. These included the surgical techniques used, trial cohort composition, number of patients per group, length of reported follow-up, hypospadias meatus location and coverage layers over urethroplasty per group. Outcome data extracted from the articles included the following variables: operative time, length of hospital stay, urethral fistula, meatal stenosis, urethral stricture, wound infection, glans dehiscence and bleeding frequencies. Two investigators independently extracted the predefined data using standardized forms. Disagreements were resolved by verification and consensus among all authors.

### 2.6. Data Synthesis

A meta-analysis for the outcomes was conducted using Review Manager (RevMan) Software v.5.3.5 and utilizing the Cochrane handbook [36]. Operative time and hospital stay were analyzed as continuous variables using the mean difference (MD) with a 95% confidence interval and the inverse-variance method. Postoperative complication outcomes, i.e., urethral fistula, meatal stenosis, urethral stricture, wound infection, glans dehiscence and bleeding/hematoma formation were analyzed as dichotomous variables using the Mantel-Haenszel fixed-effects model to calculate Risk Ratio (RR) with 95% confidence intervals. This model performs well when analyzing studies with zero events in study arms and is better suited for studies where events and sample sizes are low. Forest plots are provided per outcome. The assessment of heterogeneity was conducted using I^2^, with I^2^ < 25% indicating low heterogeneity, 25–50% moderate and >50% suggesting high heterogeneity. For outcomes where it was perceived as high, a sensitivity analysis was carried out by gradually analyzing the results after omitting studies that contributed to the high heterogeneity. Leave-one-out sensitivity analysis was performed to assess the influence of individual studies on the pooled fixed-effect (inverse-variance) estimate. Case-deletion influence diagnostics (standardized residuals, DFFITS, Cook’s distance, leverage, and covariance ratios) were computed for each study following the approach implemented in the metafor package [37]. These analyses were conducted using Research Gold (https://researchgold.org (accessed on 23 September 2026)). Continuous variables reported using medians, means and range were converted by application of formulas by Luo et al. to calculate mean and standard deviation (SD) [38]. Subgroup analyses were conducted where appropriate by primary or reoperative surgery, PRF as solitary or adjunctive layer, operative technique used and hypospadias location. A *p*-value less than 0.05 was defined as statistically significant. Descriptive statistics were used to provide a summary of interesting information to the readers where applicable. The GRADE handbook was consulted to grade the certainty of evidence [39].

### 2.7. Limitations of the Study

This review presents data from a multitude of clinical studies utilizing PRF as an autologous graft layer for both primary and re-operative hypospadias surgery in a variety of clinical settings and by several surgeons. Careful consideration should be given to the fact that there is high heterogeneity among studies because of the variety of applied techniques, their modifications, the PRF production method and the modes of application. This limits protocol reproducibility. Hypospadias surgery is a fine and delicate field where both technical prowess as well as knowledge and expertise of the surgeon are of the utmost importance. Limitations also arise due to patient factors and anatomy. This cannot be underestimated as repair of hypospadias is a perfect example of a personalized application of surgical technique. The surgeon must draw from a rich armamentarium of knowledge and skill on several procedures to tailor the approach to the individual patient intraoperatively. PRF production has not been extensively standardized and heterogeneity between preparations should be expected.

**Figure 2:**
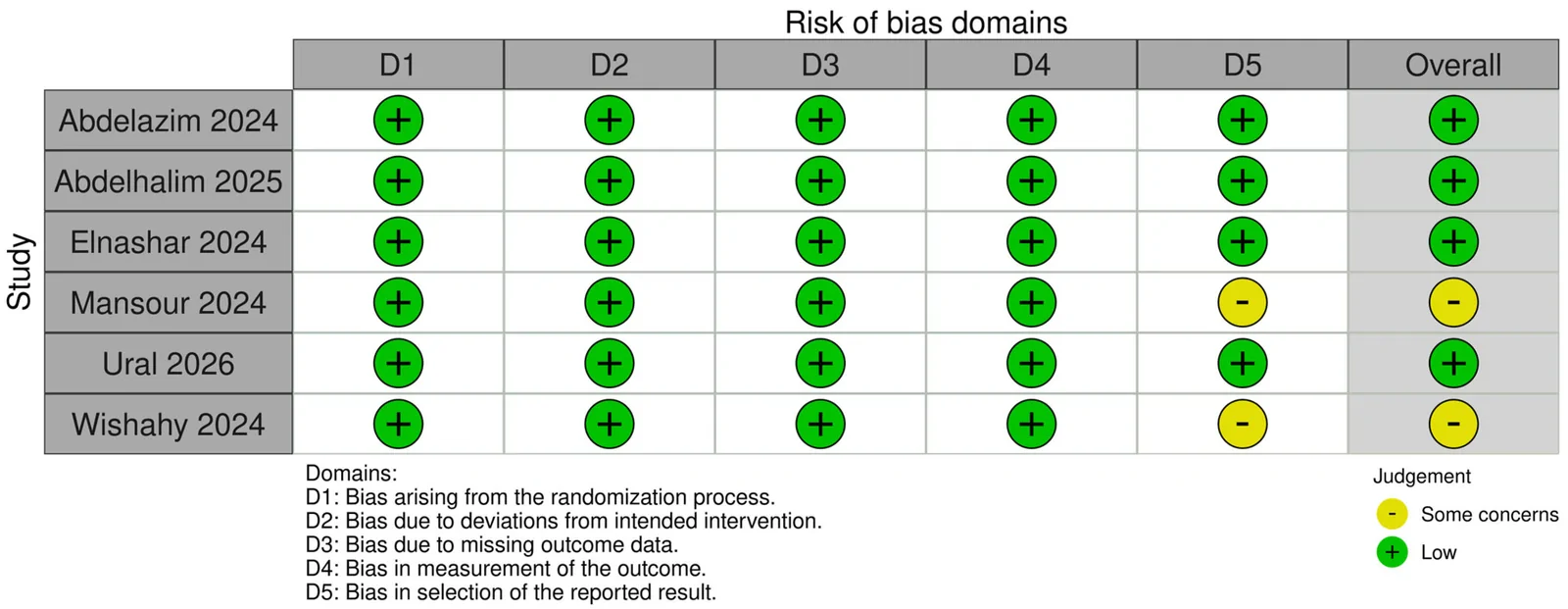
Risk of bias traffic light plot of included randomized controlled trials after assessment using RoB 2. + (green), indicates low risk of bias; - (yellow), some concerns for bias. Generated by robvis. Abdelazim 2024 [43]; Abdelhalim 2025 [44]; Elnashar 2024 [45]; Mansour 2024 [46]; Ural 2026 [47]; Wishahy 2024 [48].

**Figure 3:**
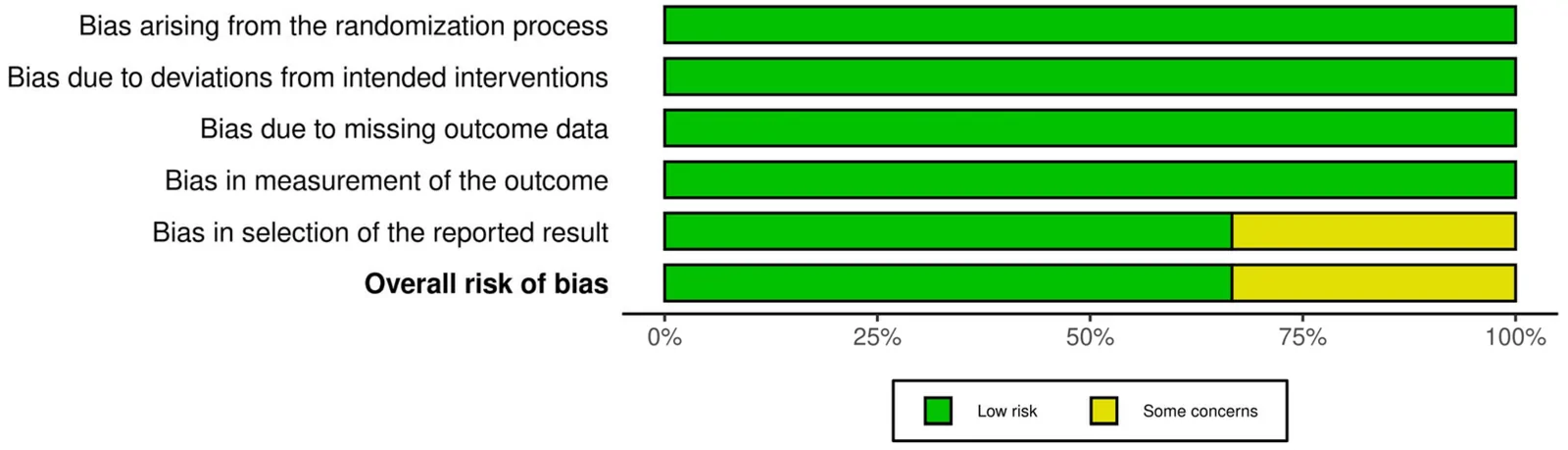
Risk of bias summary plot of included randomized controlled trials. Generated by robvis.

Assessment for risk of bias was conducted, using the RoB-2 revised tool to assess risk of bias in RCTs and the findings are presented in a traffic-light plot and a summary plot prepared by the robvis tool [40,41]. The Cochrane handbook was consulted during the risk of bias assessment [36]. The risk of bias was deemed low for 4 RCTs and there were some concerns for bias in selection of the reported result for 2 RCTs (Figure 2 and Figure 3). These concerns are raised specifically for the studies by Mansour et al. and Wishahy et al. that did not provide specific information whether unblinded outcome data were analyzed according to a pre-specified protocol before their acquisition. Minor remarks on potential sources of bias include the baseline characteristics in the study by Elnashar et al. where the PRF study group contained less coronal and more distal penile cases which may be associated with worse outcomes, but we consider the risk of bias to be minimal since these percentages can be attributed to chance during randomization. Also, in the study by Mansour et al. there was 1 case lost to follow-up after randomization that could contribute to bias in outcomes due to the low number of participants per group.

**Figure 4:**
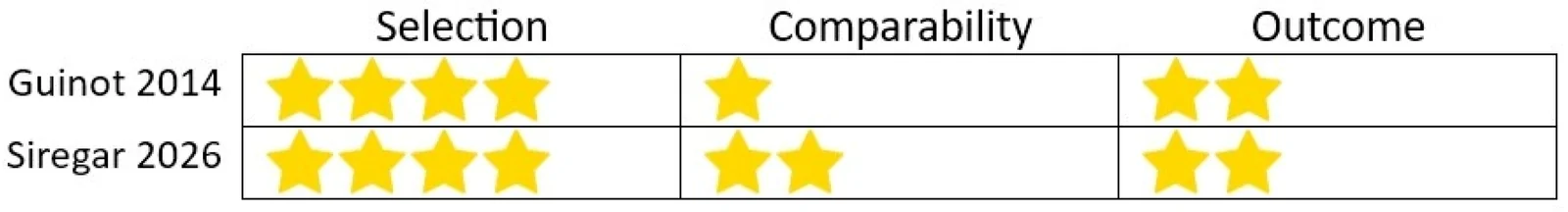
Risk of bias of included non-randomized cohort studies using the Newcastle–Ottawa quality assessment scale. A higher number of stars means a lower risk of bias assessment. Maximum number of stars: 4 for selection, 2 for comparability and 3 for outcome. Comparability of Guinot 2014 was given only one star due to the use of a historical control group. Guinot 2014 [49]; Siregar 2026 [50].

The Newcastle–Ottawa Scale was employed for quality assessment of non-randomized CCTs. The Newcastle–Ottawa Scale defines a maximum score of 9 stars across three domains of potential bias. If a study reached a score of 7 or more, it was deemed of sufficiently high quality and included in the analysis. The 2 prospective non-randomized CCTs included scored 7 and 8 stars and were thus considered of high quality and of potentially low risk for bias (Figure 4) [42].

The body of evidence presented in this systematic review and meta-analysis should help the readers appraise current progress on the use of PRF as a layer for buttressing a urethroplasty. Data on the cost of PRF membrane production were not provided in included studies, so cost-effectiveness analysis was not performed.

## 3. Results

### 3.1. Study Selection

The search of PubMed and Scopus databases provided 27 and 34 results, respectively. After deduplication, the 34 articles that remained were screened by reading the abstracts and identifying clinical trials involving pediatric hypospadias patients undergoing surgery with the use of PRF as a protective membranous layer for the repair. There were 33 selected papers for full-text appraisal. One study was unable to be retrieved. Among the excluded studies were eight articles that were letters and comments to the authors or editors, eight reviews, two articles comprising a retracted study and its retraction notice, one paper that reported a limited case series with no controls, one paper about adult patients and five articles reporting the use of PRP injections or PRP gel during surgical repair. One study was identified after reviewing the included studies’ references and it was added to this review. Finally, eight full-text studies were included that met the predefined criteria for inclusion and exclusion.

### 3.2. Study Characteristics

**Figure 5:**
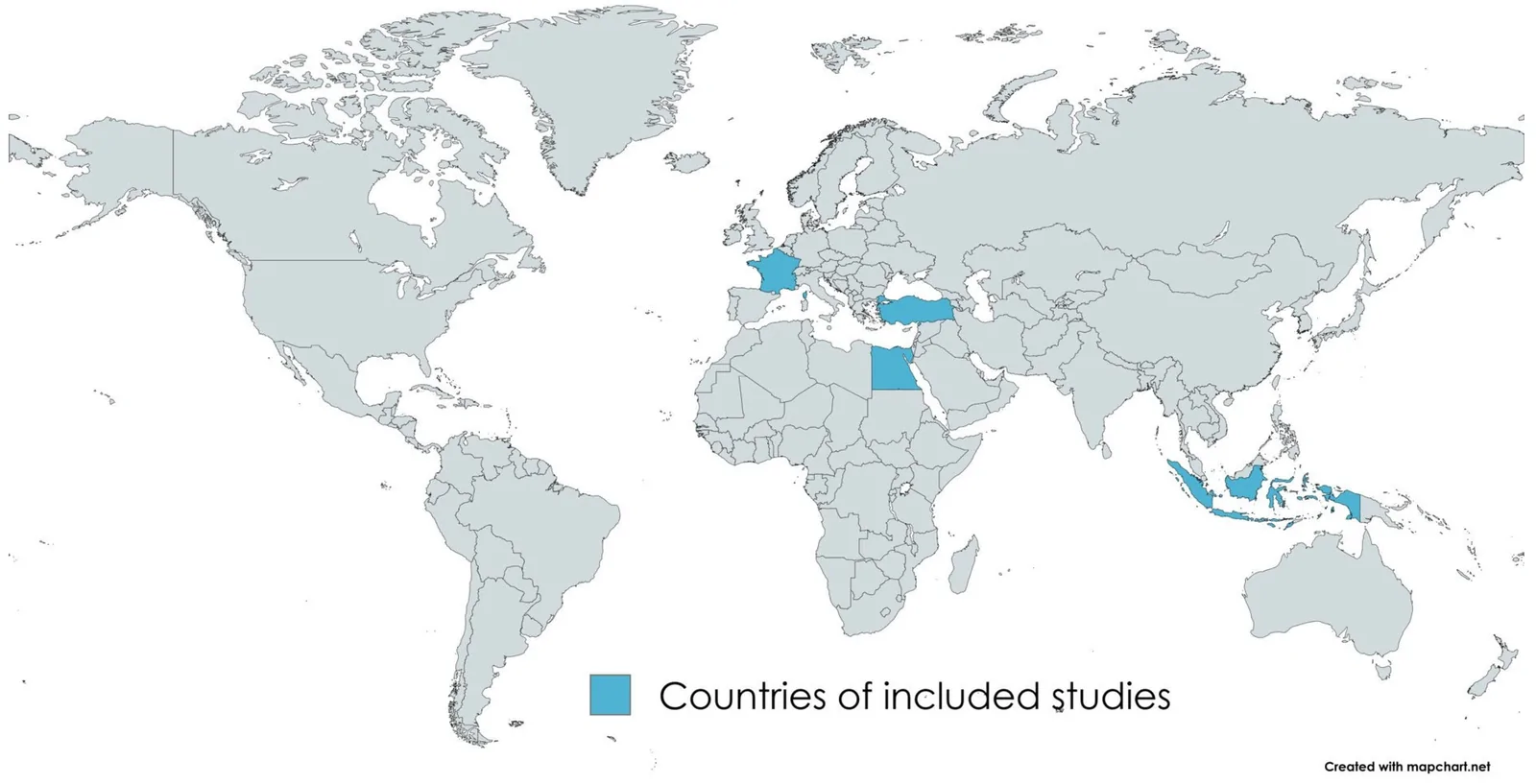
Countries of origin of included studies (map created with MapChart.net (accessed on 20 September 2026)).

Publication details are summarized in Table 1 and Figure 5. Included studies were published between 2014 and 2026, and their designs consisted of six RCTs, one prospective non-randomized controlled study and one prospective clinical study with a historical patient control group. The majority of the studies were conducted by centers in Egypt, and the rest were conducted in Turkey, France and Indonesia.

### 3.3. Patient Characteristics

The total number of reported study populations in the studies in this review was 410, with 269 patients included in six RCTs and 141 in two CCTs. The technique mostly used was TIP urethroplasty in six studies, two stage Thiersch-Duplay repair was used in one study and one study reported single or multiple fistula closure. The initial location of the urethral meatus was distal in six studies, proximal in the study by Siregar et al., while the study by Ural et al. reported on patients with distal and mid-penile hypospadias location. Wishahy et al. also reported a variable number of fistulas (single or multiple) in various locations. Two studies, namely the studies by Abdelhalim et al. and Wishahy et al., included patients with a prior failed hypospadias operation while the others included only primary cases. Six studies had a cohort with a single solitary PRF layer versus cohorts utilizing a dartos flap variant. The remaining two studies had a group with PRF as an extra adjunctive layer over a dartos flap versus groups with a single dartos flap over the urethroplasty. Age of patients was variable, but comparable between respective groups in the included articles, with most patients being infants and toddlers. Follow-up length was at least 6 months in all but two studies, namely Ural et al. and Wishahy et al. where patients were followed only for 3 months. Four studies had patients operated by a single surgeon. Patient overlap can be mostly excluded although there could be possible overlap for patients in two studies from the same center during 2022 (Abdelhalim et al. and Mansour et al.). The previously mentioned characteristics have been compiled in Table 1 and Table 2.

### 3.4. PRF Membrane Formulations

The included studies characterized the urethroplasty coverage layer as a PRF or Bio-PRF membrane. The technique for production of this membrane included a single centrifugation cycle at variable Rotations Per Minute (RPM) and variable duration of time. Relative Centrifugal Force (RCF) or g-force is dependent on the RPM and the diameter of the centrifuge, i.e., the machine used. These parameters were not always reported in the literature. Choukroun originally described PRF and the protocol for its production, now also referred to as leukocyte rich PRF or L-PRF [51]. Various other protocols yielding variations of PRF, such as advanced PRF (A-PRF), have been formally or informally used since then [34]. Notes on blood volume have been provided as venipuncture can be difficult in infants. Blood collection was mainly performed in the operating room and the PRF membrane was immediately applied after being formed. PRF should be applied within minutes of formulation as additives are not being used [34]. Table 3 summarizes the details of included studies’ PRF formulations and various production parameters. Excluded studies with PRP formulations that did not qualify as PRF membrane products [52,53,54,55,56] are provided in Supplementary Table S2.

### 3.5. Patient Outcomes

Intraoperative and postoperative outcome data are presented in the following section and are also provided in Table 4.

#### 3.5.1. Operative Time

Four of the eight included studies evaluated the total operative time required. In general, groups with PRF application slightly prolonged the operative time. Abdelhalim et al. noted a minor time increase when using a solitary PRF membrane compared to a dartos flap (107.9 ± 9.721 min vs. 102.8 ± 9.972 min), with similar results reported by Siregar et al. (151 ± 5.64 min vs. 148.67 ± 6.32 min). In the same vein, slightly extended time was noted by Mansour et al., where one group had PRF membrane applied adjunctively over a dartos flap vs. a dartos flap alone (105 ± 2.1 min vs. 90 ± 1.8 min). Conversely, Elnashar et al. found that PRF membrane application required less operative time than a ventral dartos flap (95.7 ± 11.9 min vs. 102.3 ± 10.3 min).

**Figure 6:**
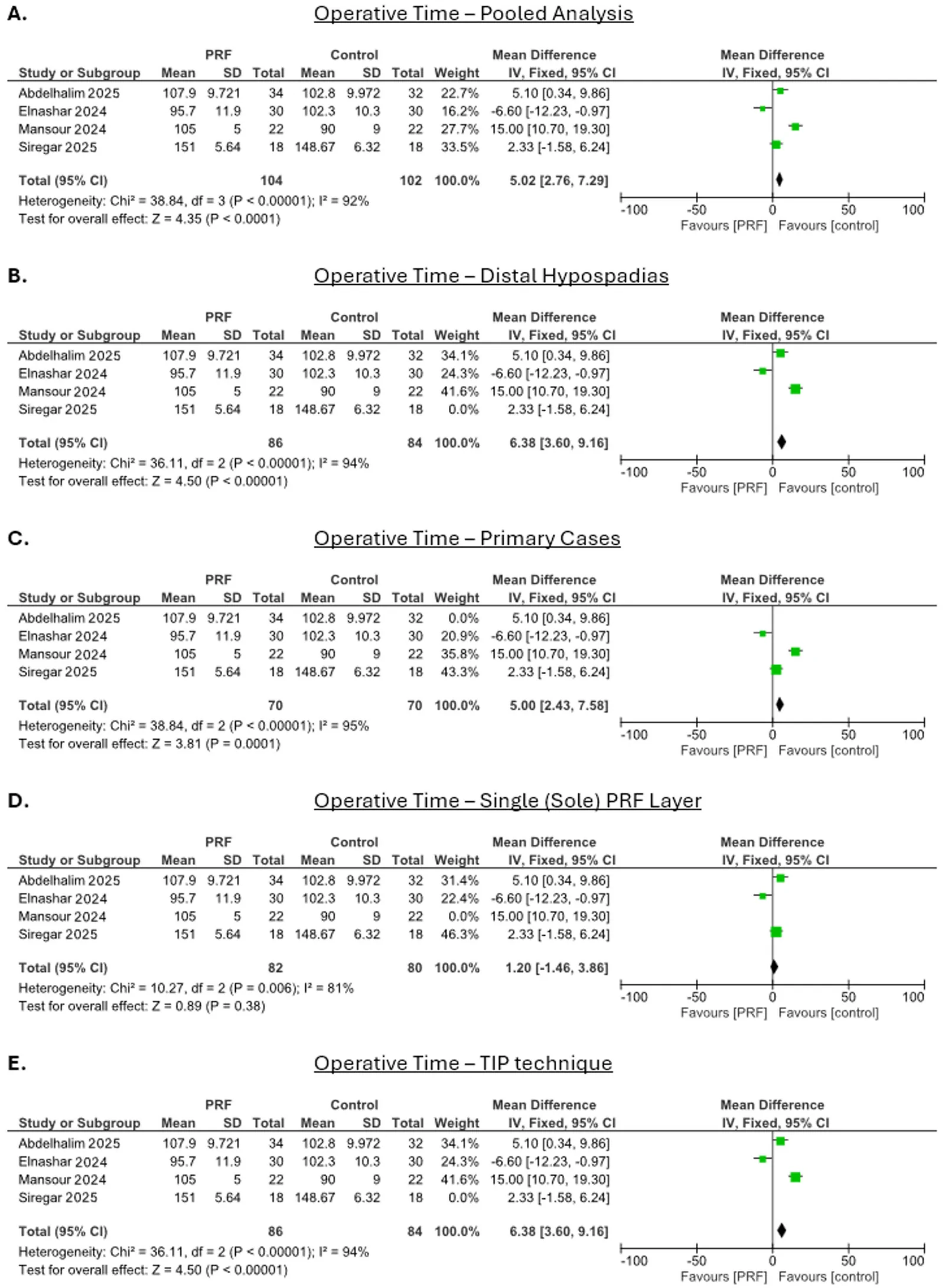
Forest plots of operative time. (**A**) pooled effect analysis of all included studies, (**B**) subgroup analysis of distal hypospadias cases, (**C**) subgroup analysis of primary hypospadias cases, (**D**) subgroup analysis of studies where a single PRF layer was used in the study group, (**E**) subgroup analysis of TIP technique cases. Abdelhalim 2025 [44]; Elnashar 2024 [45]; Mansour 2024 [46]; Siregar 2025 [50].

A fixed-effect model (inverse-variance method) was used to synthesize the results of four studies. The pooled MD was 5.02 min (95% CI: 2.76 to 7.29), indicating lower operative time for controls without PRF usage, a statistically significant finding (*p* < 0.0001). Subgroup analyses indicate similar results with statistical significance when analyzing the subgroup of distal cases (MD = 6.38–95% CI: 3.60–9.16, *p* < 0.00001), primary cases (MD = 5.00–95% CI: 2.43–7.58, *p* = 0.00001). The TIP technique subgroup comprises the same studies as the distal cases subgroup. Longer operation duration becomes less pronounced when PRF membrane is used as a single layer (MD = 1.20–95% CI: −1.46–3.86, *p* = 0.38). Heterogeneity was assessed as high using the Chi-squared Q statistic (Chi^2^ = 38.84, df = 3, *p* < 0.00001) and the I-squared statistic (I^2^ = 92%). (Figure 6).

**Figure 7:**
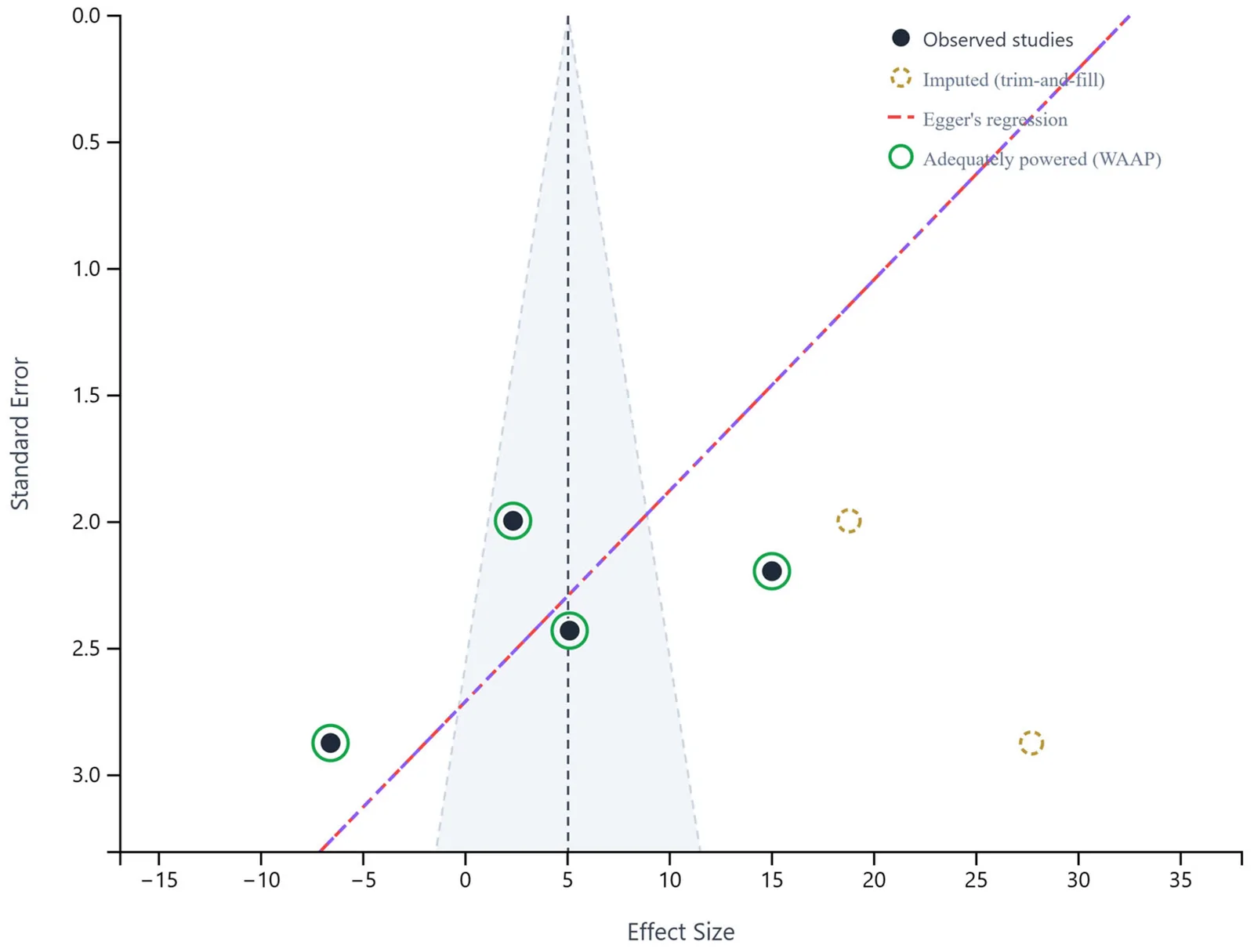
Funnel plot of included studies that report operative time.

Publication bias was assessed using a funnel plot of four studies, supplemented by Egger’s regression test and Begg’s rank correlation test [57,58]. Visual inspection of the funnel plot showed no substantial asymmetry, consistent with the results of Egger’s test (intercept = −12.00, t(2) = −0.82, *p* = 0.497) and Begg’s test (tau = −0.333, *p* = 0.734). Duval and Tweedie’s trim-and-fill procedure imputed two missing studies, yielding an adjusted pooled estimate of 10.551 (95% CI: 8.701 to 12.402), compared with the unadjusted estimate of 5.024 [59]. WAAP-WLS analysis was used as an additional bias-correction method [60,61]. The WLS estimate was 32.508 (95% CI: −33.579 to 98.595). Four of four studies were adequately powered (SE ≤ 38.626) to detect the WLS-adjusted effect at 80% power. The WAAP estimate, based on the inverse-variance weighted average of these adequately powered studies, was 5.024 (95% CI: 2.760 to 7.287) (Figure 7).

**Figure 8:**
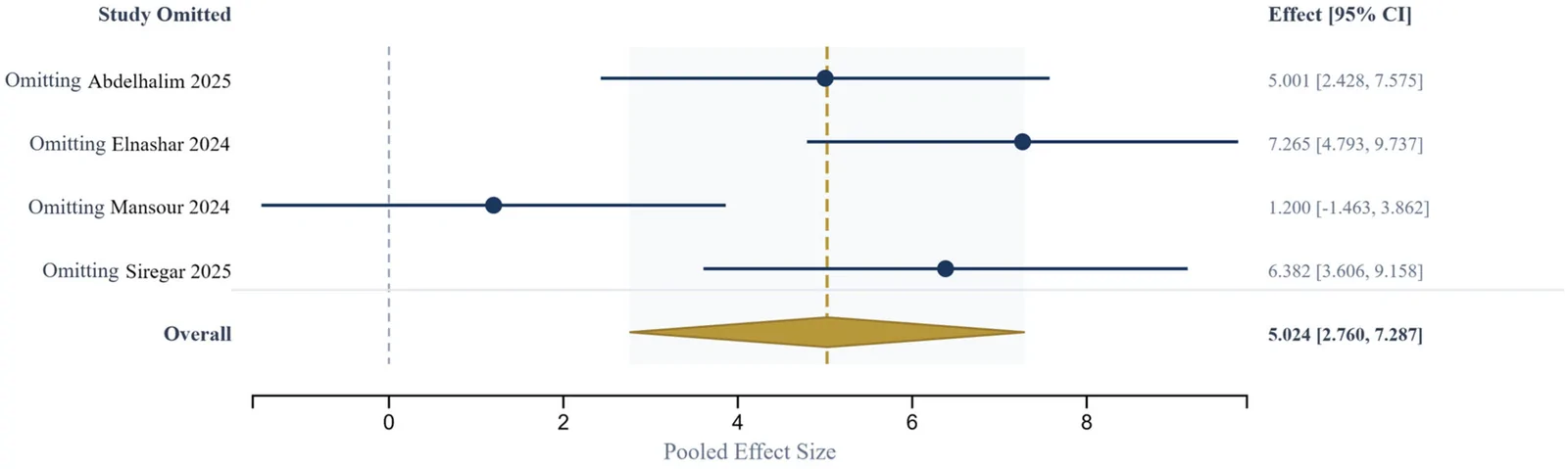
Leave-one-out sensitivity analysis plot of operative time. Abdelhalim 2025 [44]; Elnashar 2024 [45]; Mansour 2024 [46]; Siregar 2025 [50].

During sensitivity analysis two studies were flagged as potentially influential (Elnashar et al., Mansour et al.) based on standard cutoffs (|standardized residual| > 1.96, DFFITS > 3 × sqrt(1/(k − 1)), or leverage > 3 times the mean). Mansour et al. had the largest influence, shifting the pooled estimate from 5.024 to 1.200 when removed, which also changed the statistical significance of the result. Removal of this study changed the statistical significance of the pooled estimate (Figure 8).

#### 3.5.2. Hospital Stay

Length of hospital stay was reported in three studies, with no significant differences between groups. Abdelhalim et al. observed nearly identical hospital stays between groups (4.81 ± 1.575 days for dartos layer vs. 4.82 ± 1.678 days for the PRF group). In the trial by Elnashar et al., both the PRF and the ventral dartos flap cohorts exhibited an identical mean length of stay of 5.5 days (±1.1 and ±0.9 days, respectively). Furthermore, Mansour et al. reported the same hospitalization periods averaging exactly 4 ± 0.4 days.

**Figure 9:**
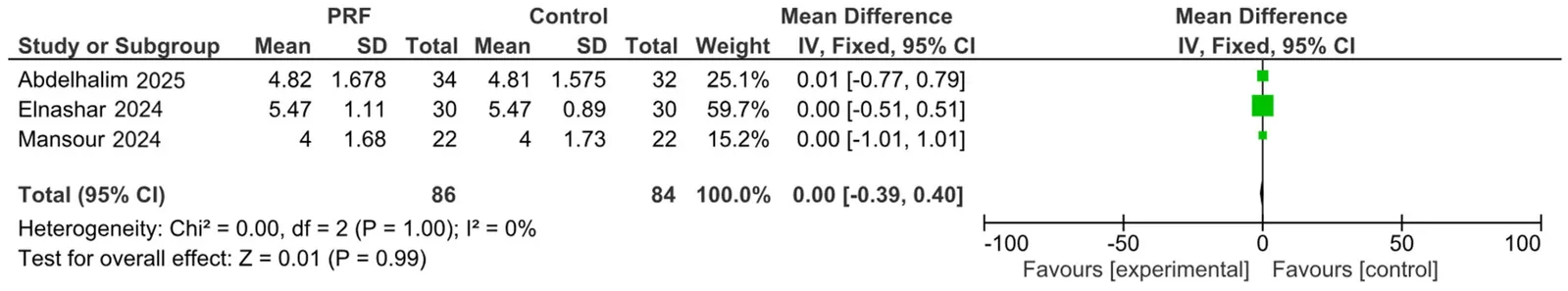
Forest plot of length of hospital stay. Abdelhalim 2025 [44]; Elnashar 2024 [45]; Mansour 2024 [46].

A fixed-effect model (inverse-variance method) was used to synthesize the results of three studies. The pooled MD was 0.00 (95% CI: −0.39 to 0.40, *p* = 0.99). Heterogeneity was assessed using Chi-squared statistic (Chi^2^ = 0.00, df = 2, *p* = 1.000) and the I-squared statistic (I^2^ = 0.0%). There was no heterogeneity among studies (Figure 9).

**Figure 10:**
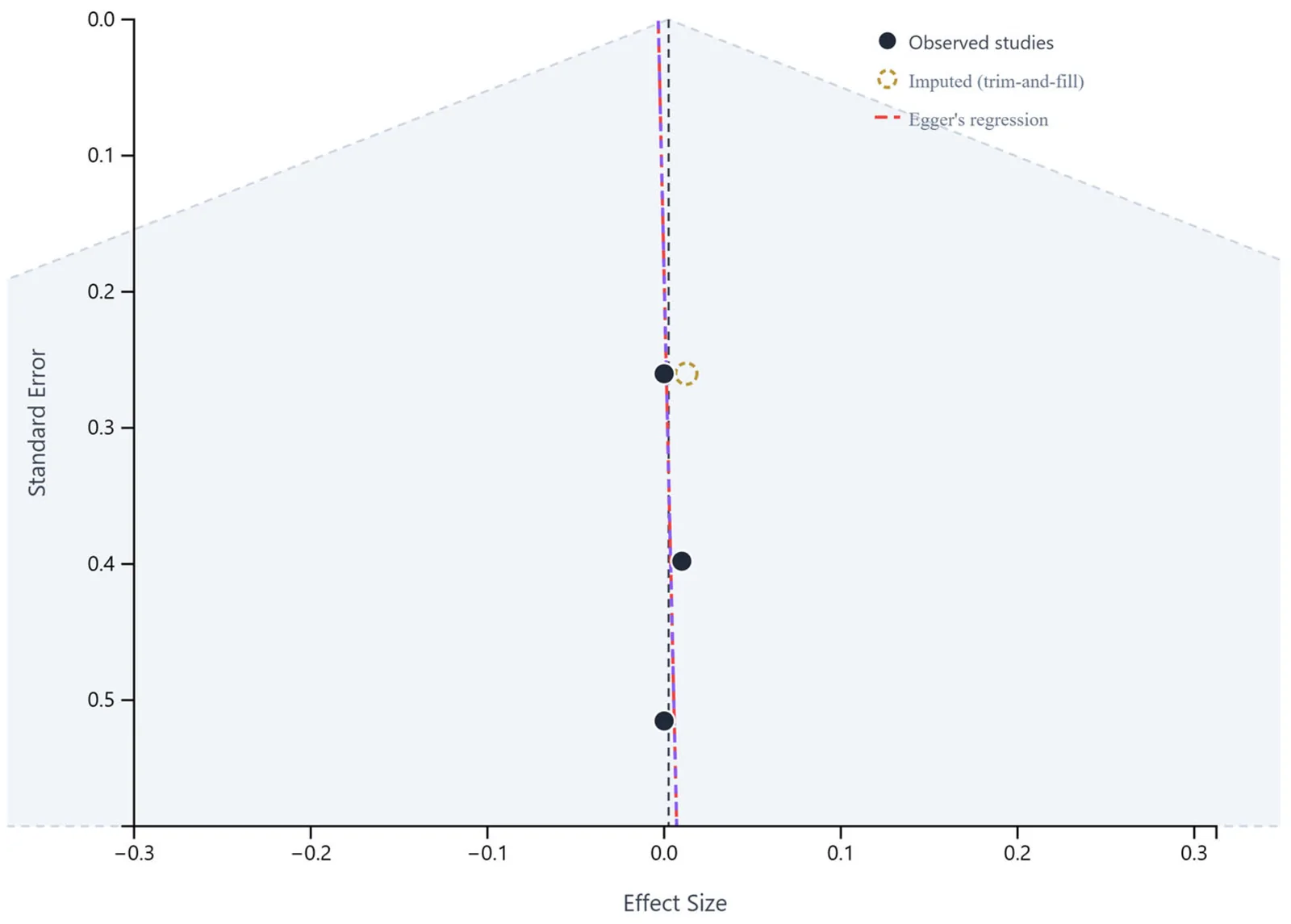
Funnel plot of included studies that report hospital stay.

Publication bias was assessed using a funnel plot of three studies, supplemented by Egger’s regression test and Begg’s rank correlation test. Visual inspection of the funnel plot showed no substantial asymmetry, consistent with the results of Egger’s test (intercept = 0.02, t(1) = 0.42, *p* = 0.746) and Begg’s test (tau = 0.000, *p* = 1.000). Duval and Tweedie’s trim-and-fill procedure imputed one missing study, yielding an adjusted pooled estimate of 0.006 (95% CI: −0.305 to 0.318), compared with the unadjusted estimate of 0.003. WAAP-WLS analysis was used as an additional bias-correction method. The WLS estimate was −0.003 (95% CI: −0.032 to 0.025). No studies met the adequacy threshold (SE ≤ 0.004) for 80% power to detect the WLS-adjusted effect, so the WLS estimate was used as a fallback (Figure 10).

#### 3.5.3. Urethral Fistula

Postoperative urethral fistula appearance was reported across all eight included trials. Abdelhalim et al. documented six fistulas in the dartos flap arm and one in the PRF arm (18.75% vs. 2.94%). Reduction in fistula occurence were also visible in the series by Abdelazim et al. (2 cases or 10% for PRF that closed spontaneously hence zero cases vs. 7 cases or 35% for preputial/dartos flap), Mansour et al. (1 for pedicled dartos flap plus PRF vs. 4 for pedicled dartos flap—4.54% vs. 18.18%), Ural et al. (0 for ventral dartos flap + PRF vs. 2 for ventral dartos flap only—4.54% vs. 27.27%), and Wishahy et al. (2 for PRF vs. 6 for dartos flap—11.76% vs. 30%). An important fact to consider is that two of these studies included failed or re-do cases, namely Abdelhalim et al. and Wishahy et al. In contrast, Elnashar et al. recorded a lower incidence in the ventral dartos flap cohort (1 case—3.33%) than the standalone PRF cohort (3 cases—10%), while Siregar et al. reported no significant differences between cohorts, with 10 fistulas (55.55%) occurring in their Bio-PRF group versus 9 (50%) in the standard dartos group.

**Figure 11:**
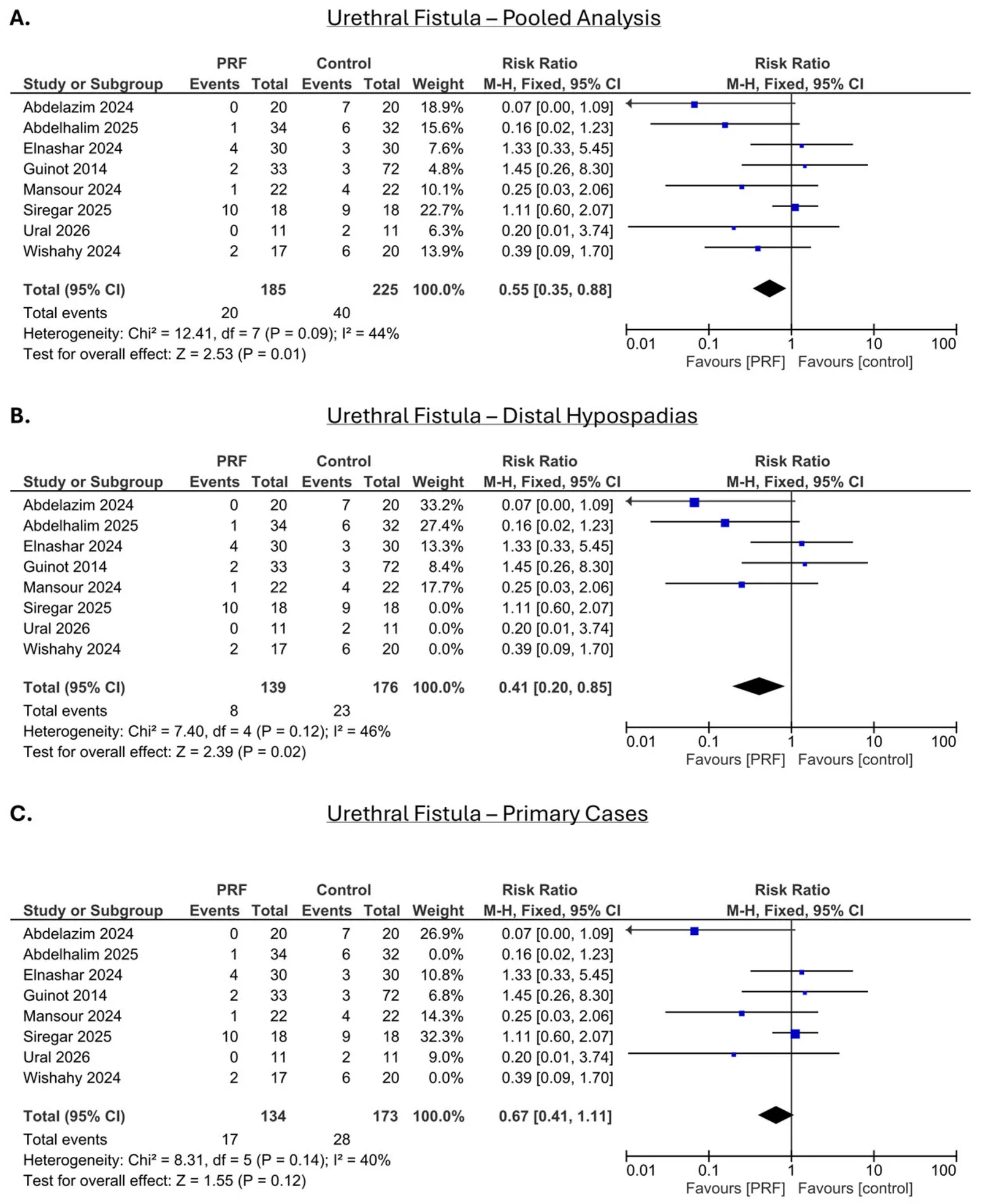
Forest plots of urethral fistula: (**A**) pooled effect analysis of all included studies, (**B**) subgroup analysis of distal hypospadias cases, (**C**) subgroup analysis of primary hypospadias cases. Abdelazim 2024 [43]; Abdelhalim 2025 [44]; Elnashar 2024 [45]; Guinot [49]; Mansour 2024 [46]; Siregar 2025 [50], Ural 2026 [47], Wishahy 2024 [48].

**Figure 12:**
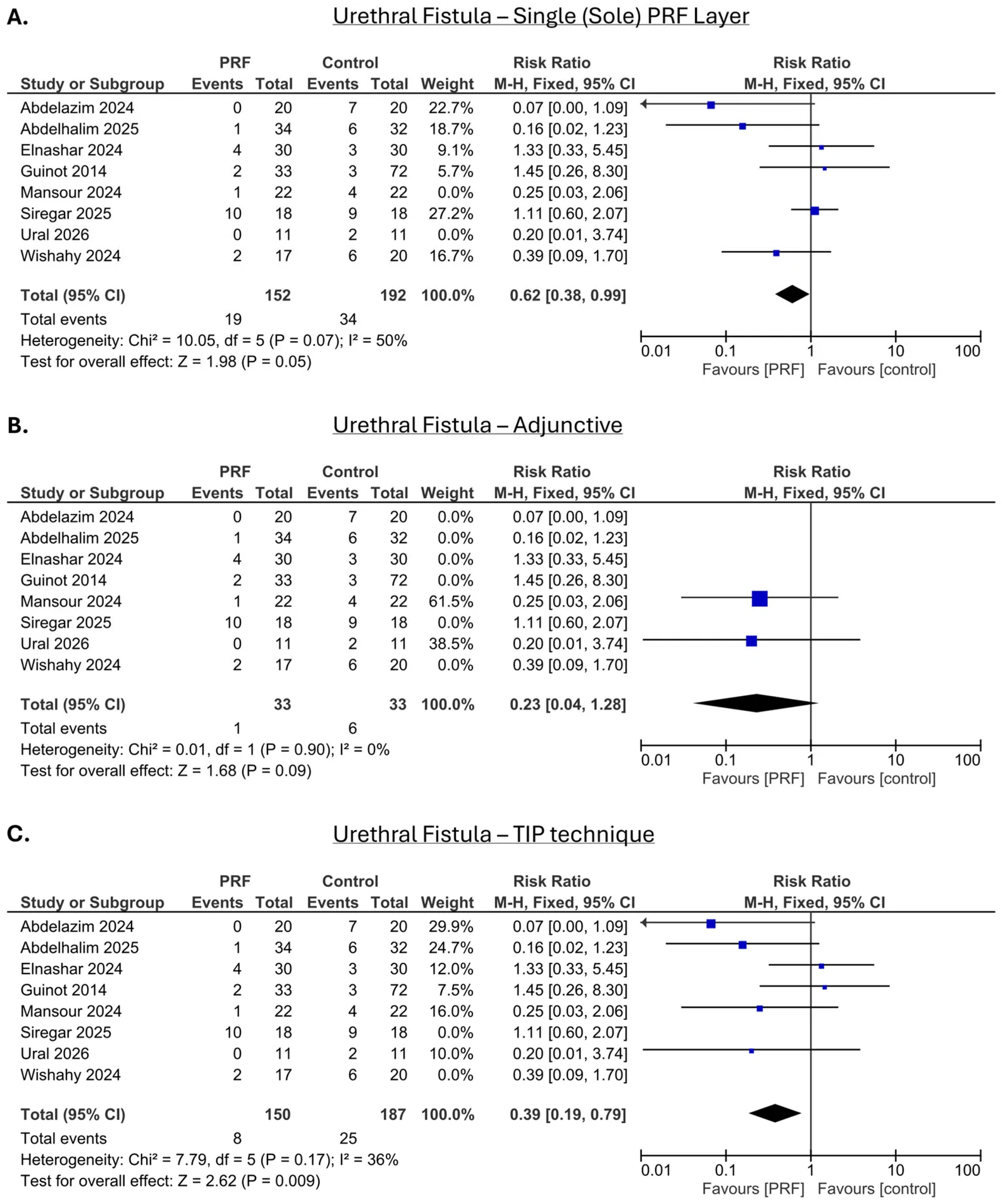
Forest plots of urethral fistula (continued), (**A**) subgroup analysis of studies where a single PRF layer was used in the study group, (**B**) subgroup analysis of studies where a PRF layer was used as an extra layer over another layer in the study group, (**C**) subgroup analysis of TIP technique cases. Abdelazim 2024 [43]; Abdelhalim 2025 [44]; Elnashar 2024 [45]; Guinot [49]; Mansour 2024 [46]; Siregar 2025 [50], Ural 2026 [47], Wishahy 2024 [48].

A fixed-effect Mantel-Haenszel method was used to synthesize the results of eight studies. Effect sizes (RR) were pooled on the natural-log scale and back-transformed for reporting. The pooled RR was 0.55 (95% CI: 0.35 to 0.88), exhibiting a statistically significant advantage when a PRF membrane is used (*p* = 0.01). Subgroup analyses also provide similar results favoring the use of a PRF membrane layer with statistical significance when analyzing the subgroups of distal cases (RR = 0.41–95% CI: 0.20–0.85, *p* = 0.02), TIP technique (RR = 0.39–95% CI: 0.19–0.79, *p* = 0.009) and solitary PRF layer usage (RR = 0.62–95% CI: 0.38–0.99, *p* = 0.05). Favor in application of PRF membrane is presented among subgroups of primary cases (RR = 0.67–95% CI: 0.41–1.11, *p* = 0.12) and adjunctive PRF membrane layer usage, albeit with no statistical significance. Heterogeneity was assessed as moderate using Chi-squared statistic (Chi^2^ = 12.41, df = 7, *p* = 0.09) and the I-squared statistic (I^2^ = 44%) for the pooled group and was also moderate among subgroups (Figure 11 and Figure 12). Siregar et al. studied proximal cases and Wishahy et al. fistula closure which are more heterogenous compared to the other included studies.

**Figure 13:**
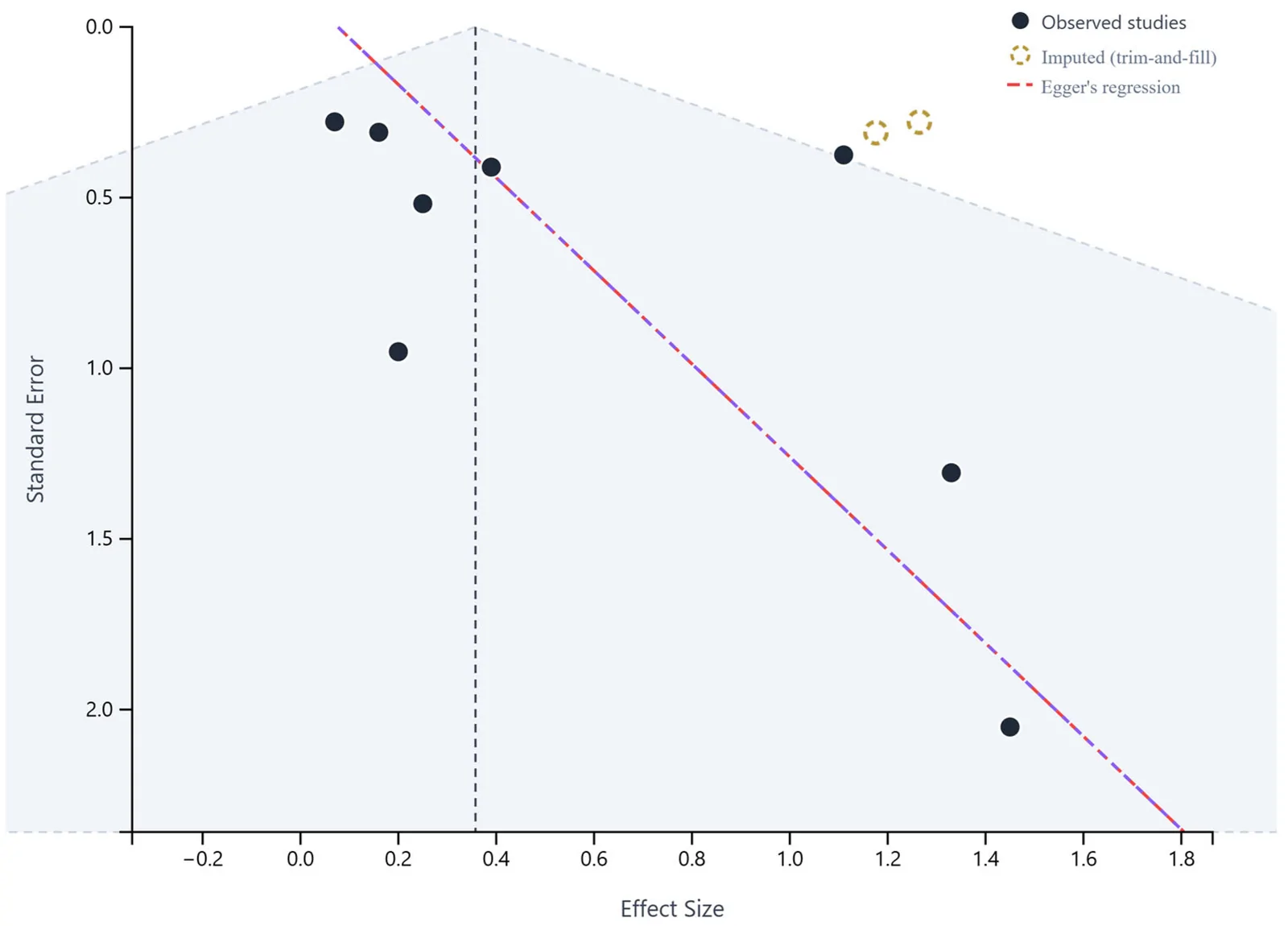
Funnel plot of included studies that report urethral fistula.

Publication bias was assessed using a funnel plot of eight studies, supplemented by Egger’s regression test and Begg’s rank correlation test. Visual inspection of the funnel plot showed no substantial asymmetry, consistent with the results of Egger’s test (intercept = 0.73, t(6) = 1.03, *p* = 0.343) and Begg’s test (tau = 0.571, *p* = 0.063). Duval and Tweedie’s trim-and-fill procedure imputed two missing studies, yielding an adjusted pooled estimate of 0.668 (95% CI: 0.426 to 0.910), compared with the unadjusted estimate of 0.358. WAAP-WLS analysis was used as an additional bias-correction method. The WLS estimate was 0.076 (95% CI: −0.532 to 0.685). No studies met the adequacy threshold (SE ≤ 0.091) for 80% power to detect the WLS-adjusted effect, so the WLS estimate was used as a fallback (Figure 13).

**Figure 14:**
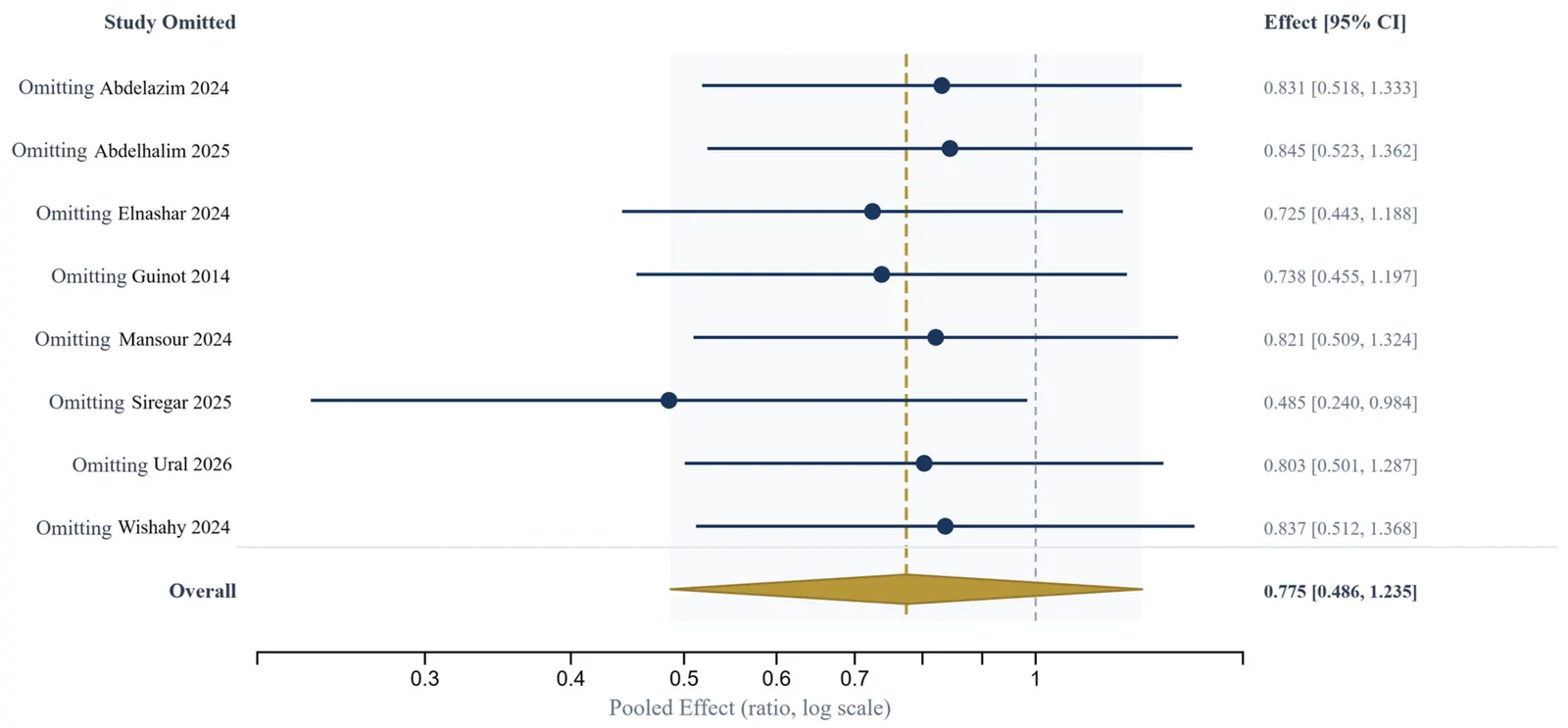
Leave-one-out sensitivity analysis plot of urethral fistula. Abdelazim 2024 [43]; Abdelhalim 2025 [44]; Elnashar 2024 [45]; Guinot [49]; Mansour 2024 [46]; Siregar 2025 [50], Ural 2026 [47], Wishahy 2024 [48].

**Figure 15:**
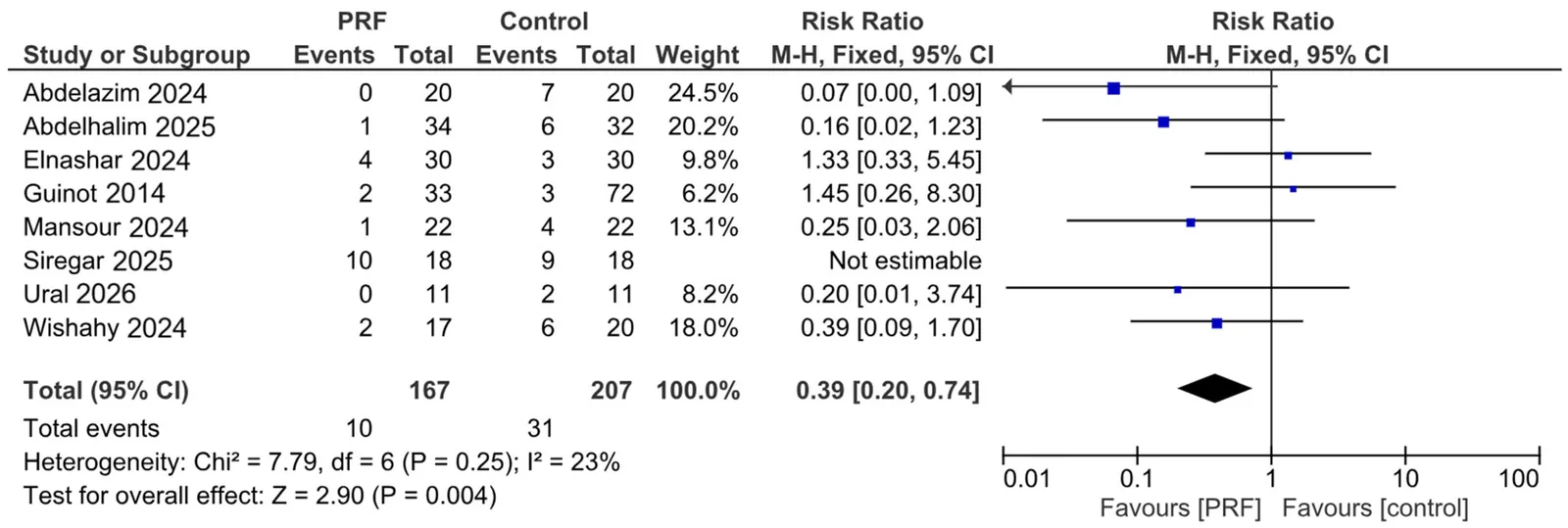
Forest plot of included studies reporting urethral fistula after omitting Siregar et al. Abdelazim 2024 [43]; Abdelhalim 2025 [44]; Elnashar 2024 [45]; Guinot [49]; Mansour 2024 [46]; Siregar 2025 [50], Ural 2026 [47], Wishahy 2024 [48].

Sensitivity analysis flagged one study as potentially influential (Siregar et al.) based on standard cutoffs (|standardized residual| > 1.96, DFFITS > 3 × sqrt(1/(k − 1)), or leverage > 3 times the mean). Siregar et al. had the largest influence, shifting the pooled estimate from 0.775 to 0.485 when removed, which also changed the statistical significance of the result. Removal of one study (Siregar et al.) changed the statistical significance of the pooled estimate. However, it remained statistically significant in favor of the PRF group (RR = 0.39–95% CI: 0.20–0.74, *p* = 0.004) with less heterogeneity (I^2^ = 23%) as expected since Siregar studied only proximal cases (Figure 14 and Figure 15).

#### 3.5.4. Meatal Stenosis

Six clinical trials provided results for meatal stenosis. In several cohorts, the integration of a PRF layer correlated with a lower incidence of meatal narrowing. Abdelhalim et al. noted a reduction from four cases in the dartos group to one case in the PRF group (12.5% vs. 2.94%). Abdelazim et al. documented a lower rate of meatal stenosis when combining a dartos flap with PRF relative to a single dartos flap (1 vs. 2 cases—5% vs. 10%). Conversely, some studies reported higher or equivalent stenosis rates when using PRF; Siregar et al. observed one meatal stenosis or 5.55% in the PRF cohort compared to zero in the dartos group. However, only Elnashar et al. noted higher meatal stenosis rates between a solitary PRF layer (7 cases—23.33%) and a ventral dartos flap (2 cases—6.66%). Guinot et al. reported zero instances of stenosis in both their PRF and dorsal foreskin subcutaneous flap arms, and Mansour et al. reported an equal distribution of two cases per group (9.09%).

**Figure 16:**
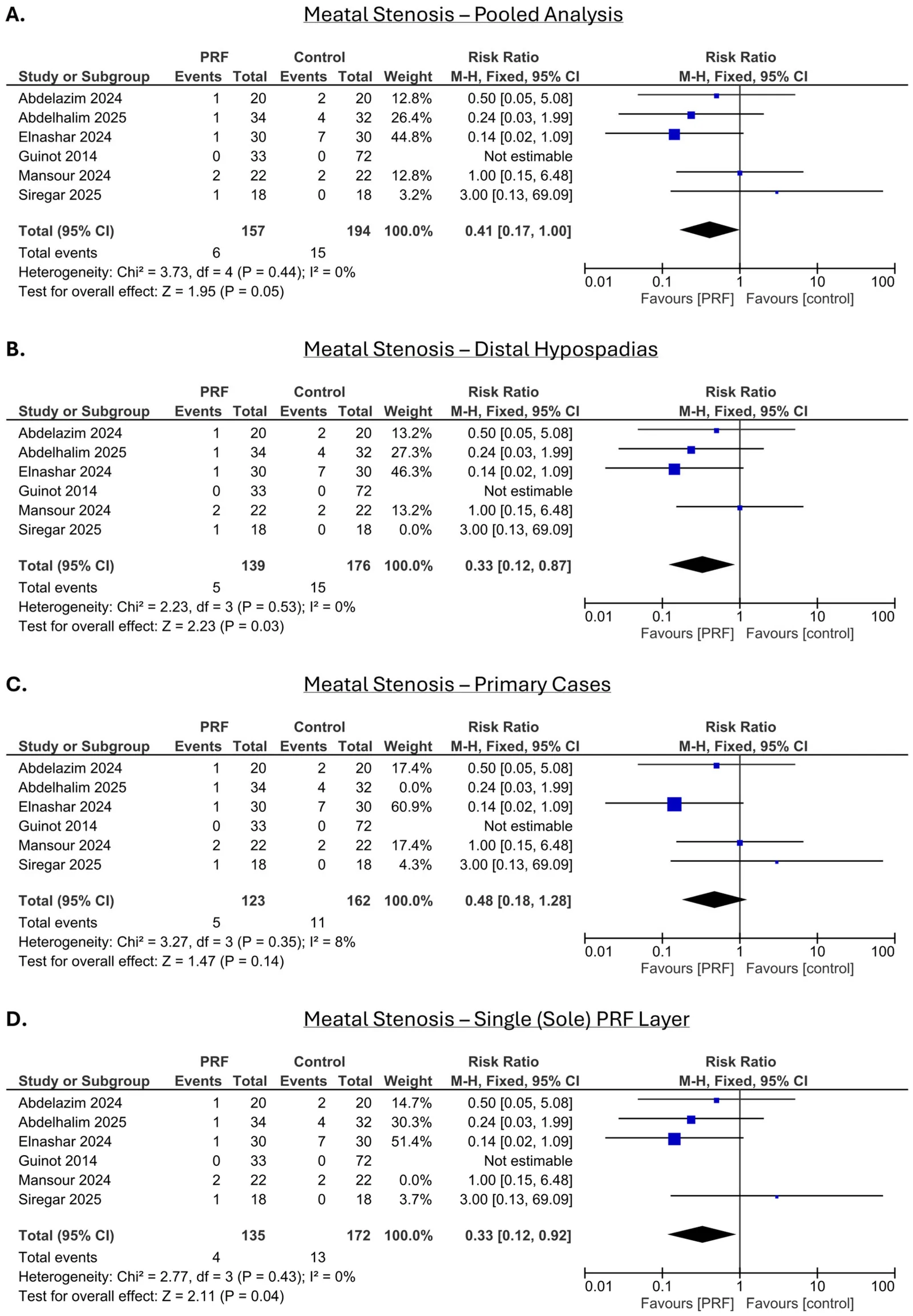
Forest plots of meatal stenosis: (**A**) pooled effect analysis of all included studies, (**B**) subgroup analysis of distal hypospadias cases (also the TIP subgroup), (**C**) subgroup analysis of primary hypospadias cases, (**D**) subgroup analysis of studies where a single PRF layer was used in the study group. Abdelazim 2024 [43]; Abdelhalim 2025 [44]; Elnashar 2024 [45]; Guinot [49]; Mansour 2024 [46]; Siregar 2025 [50].

The results of six studies were analyzed. The pooled RR was 0.41 (95% CI: 0.17 to 1.00, *p* = 0.05), suggesting a reduction in meatal stenosis for the PRF group, however with a 95% CI approaching 1.00. Subgroup analysis presented statistically significant reduction in meatal stenosis events for distal cases (RR = 0.33–95% CI: 0.12–0.87, *p* = 0.03) and single layer subgroups (RR = 0.33–95% CI: 0.12–0.92, *p* = 0.04) with the primary case subgroup having a less pronounced effect and wider CI crossing the RR point of 1.00 (RR = 0.48–95% CI: 0.18–1.28, *p* = 0.14). The TIP tehnique subgroup is the same as the distal cases subgroup. Heterogeneity was assessed as low using Chi-squared statistic (Chi^2^ = 3.73, df = 4, *p* = 0.44) and the I-squared statistic (I^2^ = 0%) (Figure 16).

**Figure 17:**
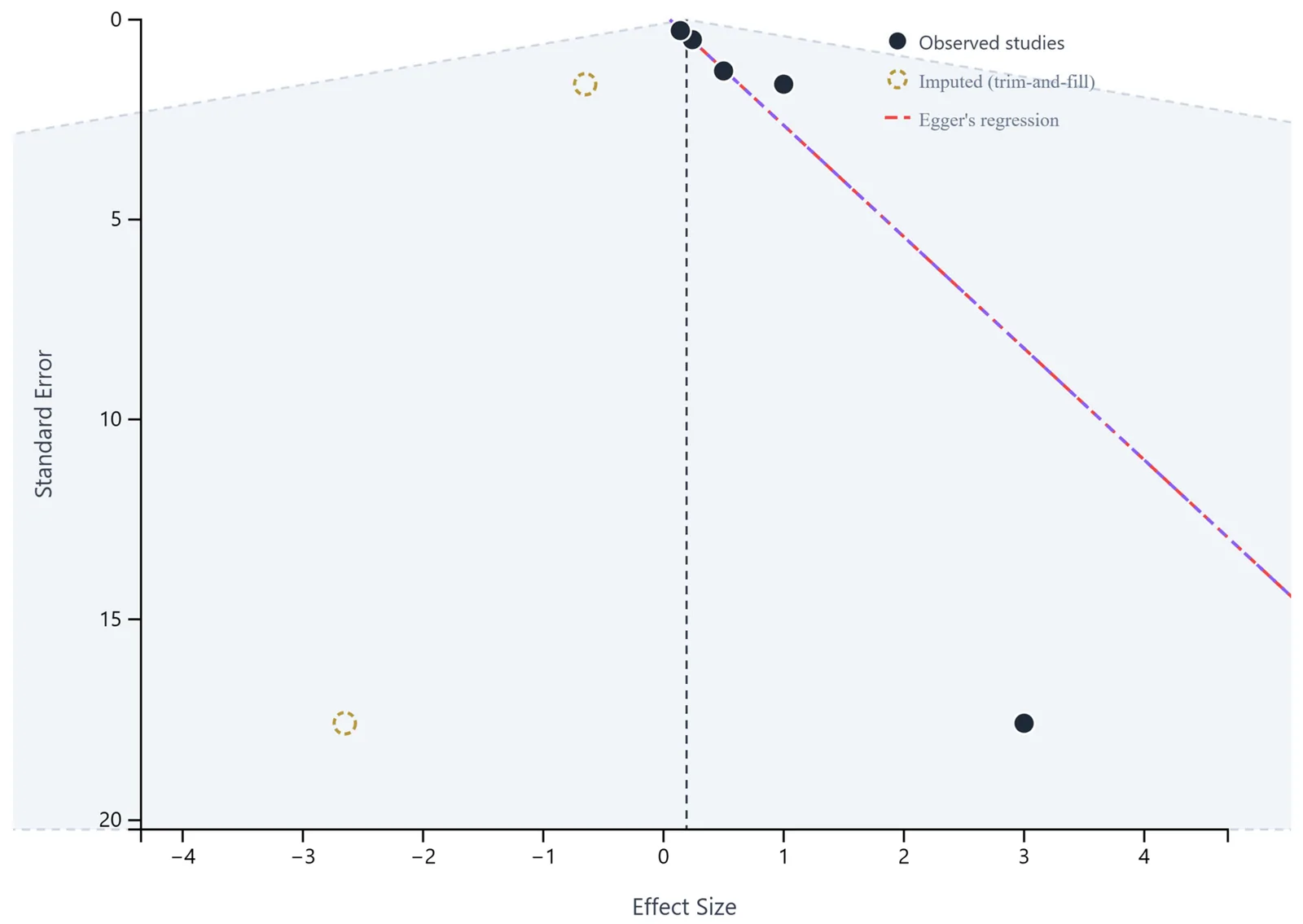
Funnel plot of included studies that report meatal stenosis.

A funnel plot of five studies, supplemented by Egger’s regression test and Begg’s rank correlation test was used to evaluate publication bias. Visual inspection of the funnel plot revealed asymmetry, consistent with the results of Egger’s test (intercept = 0.36, t(3) = 3.10, *p* = 0.053) and Begg’s test (tau = 1.000, *p* = 0.027). Duval and Tweedie’s trim-and-fill procedure imputed two missing studies, yielding an adjusted pooled estimate of 0.174 (95% CI: −0.278 to 0.626), compared with the unadjusted estimate of 0.192. WAAP-WLS analysis was used as an additional bias-correction method. The WLS estimate was 0.053 (95% CI: −0.065 to 0.172). No studies met the adequacy threshold (SE ≤ 0.064) for 80% power to detect the WLS-adjusted effect, so the WLS estimate was used as a fallback (Figure 17).

**Figure 18:**
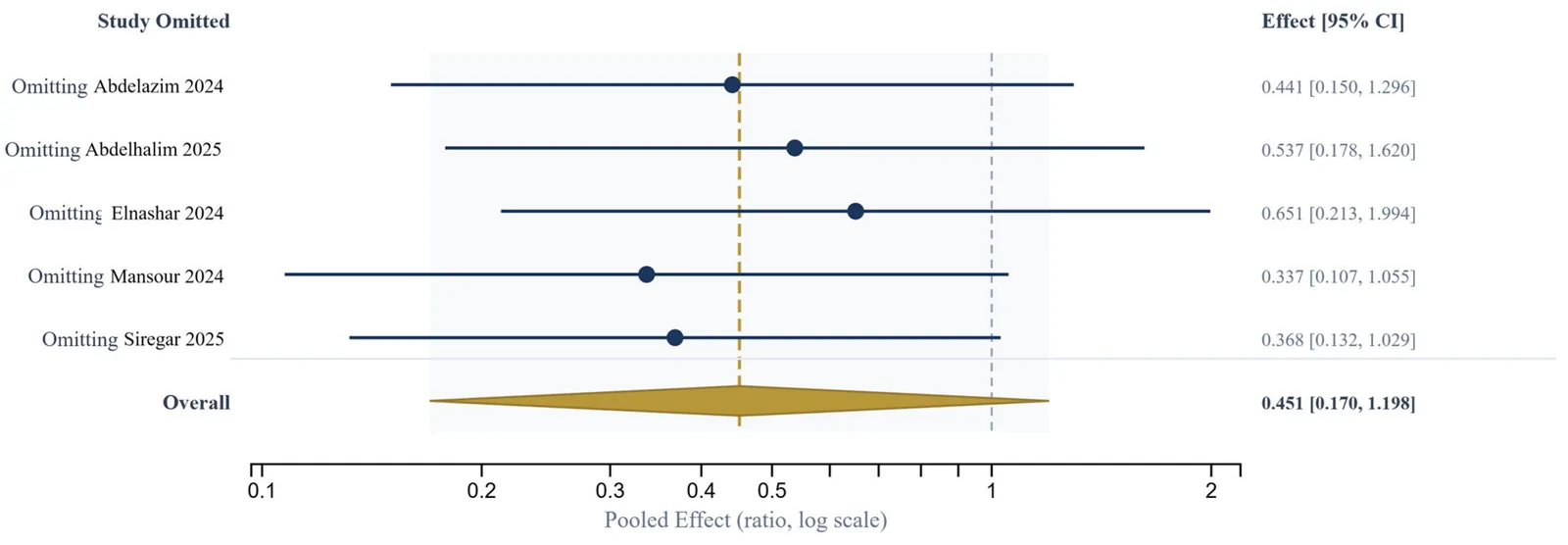
Leave-one-out sensitivity analysis plot of meatal stenosis. Abdelazim 2024 [43]; Abdelhalim 2025 [44]; Elnashar 2024 [45]; Guinot [49]; Mansour 2024 [46]; Siregar 2025 [50].

Leave-one-out sensitivity analysis was performed to assess the influence of individual studies on the pooled fixed-effect (inverse-variance) estimate. Case-deletion influence diagnostics (standardized residuals, DFFITS, Cook’s distance, leverage, and covariance ratios) were computed for each study following the approach implemented in the metafor package. No study exceeded the standard influence cutoffs. Elnashar et al. had the largest influence, shifting the pooled estimate from 0.451 to 0.651 when removed. No single study removal altered the significance or direction of the pooled estimate (95% CI: 0.170 to 1.198), indicating robust results (Figure 18).

#### 3.5.5. Urethral Stricture

Stricture of the neourethra was evaluated during follow-up in three studies, with all of them, namely the studies by Elnashar et al., Guinot et al., and Mansour et al. reporting zero urethral strictures. Meta-analysis was not conducted as this outcome was not estimable.

#### 3.5.6. Wound Infection

Wound infection rates were tracked across five trials. Abdelhalim et al. recorded a decline in infection cases, with five cases in the dartos group versus three cases in the solitary PRF group (8.82% vs. 15.62%). Fewer wound infection events were also noted by Mansour et al. (0 cases with dartos flap plus PRF vs. 5 or 22.72% of cases with a solitary pedicled dartos flap). Ural et al. corroborated this pattern, reporting no wound infection in their dartos flap with PRF cohort compared to one in the standalone dartos cohort (9.09%). Meanwhile, Abdelazim et al. reported zero wound infections for both groups and Siregar et al. noted an identical distribution of two infections in both the dartos flap and Bio-PRF study arms (11.11%).

**Figure 19:**
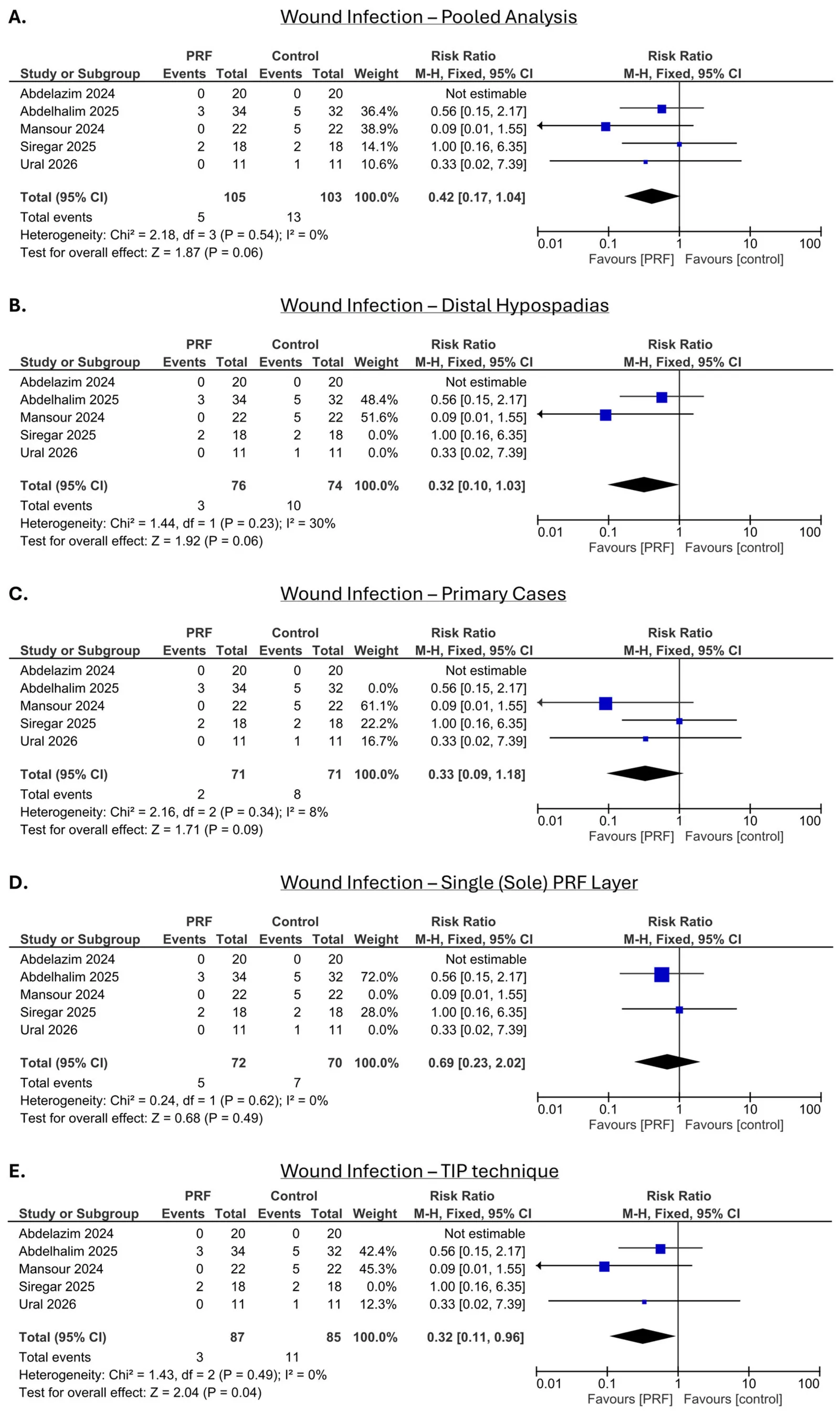
Forest plots of wound infection: (**A**) pooled effect analysis of all included studies, (**B**) subgroup analysis of distal hypospadias cases, (**C**) subgroup analysis of primary hypospadias cases, (**D**) subgroup analysis of studies where a single PRF layer was used in the study group, (**E**) subgroup analysis of TIP technique cases. Abdelazim 2024 [43]; Abdelhalim 2025 [44]; Mansour 2024 [46]; Siregar 2025 [50], Ural 2026 [47].

A fixed-effect Mantel-Haenszel method was used to synthesize the results of five studies. The pooled RR was 0.42 (95% CI: 0.17 to 1.04, *p* = 0.06), indicating lower wound infection rates for the PRF group, however with a 95% upper CI bound of 1.04. Subgroup analysis showed reduction in wound infection rates for distal cases (RR = 0.32–95% CI: 0.10–1.03, *p* = 0.06) and primary case subgroups (RR = 0.33–95% CI: 0.09–1.18, *p* = 0.09) with the single layer subgroup having a higher RR and a much wider CI crossing the RR point of 1.00 (RR = 0.69–95% CI: 0.23–2.02, *p* = 0.49). The TIP tehnique subgroup showed statistically significant less wound infections when PRF membrane was used as a coverage layer (RR = 0.32–95% CI: 0.11–0.96, *p* = 0.04). Heterogeneity was assessed as moderate using Chi-squared statistic (Chi^2^ = 1.44, df = 1, *p* = 0.23) and the I-squared statistic (I^2^ = 30%), and low among subgroups (Figure 19).

**Figure 20:**
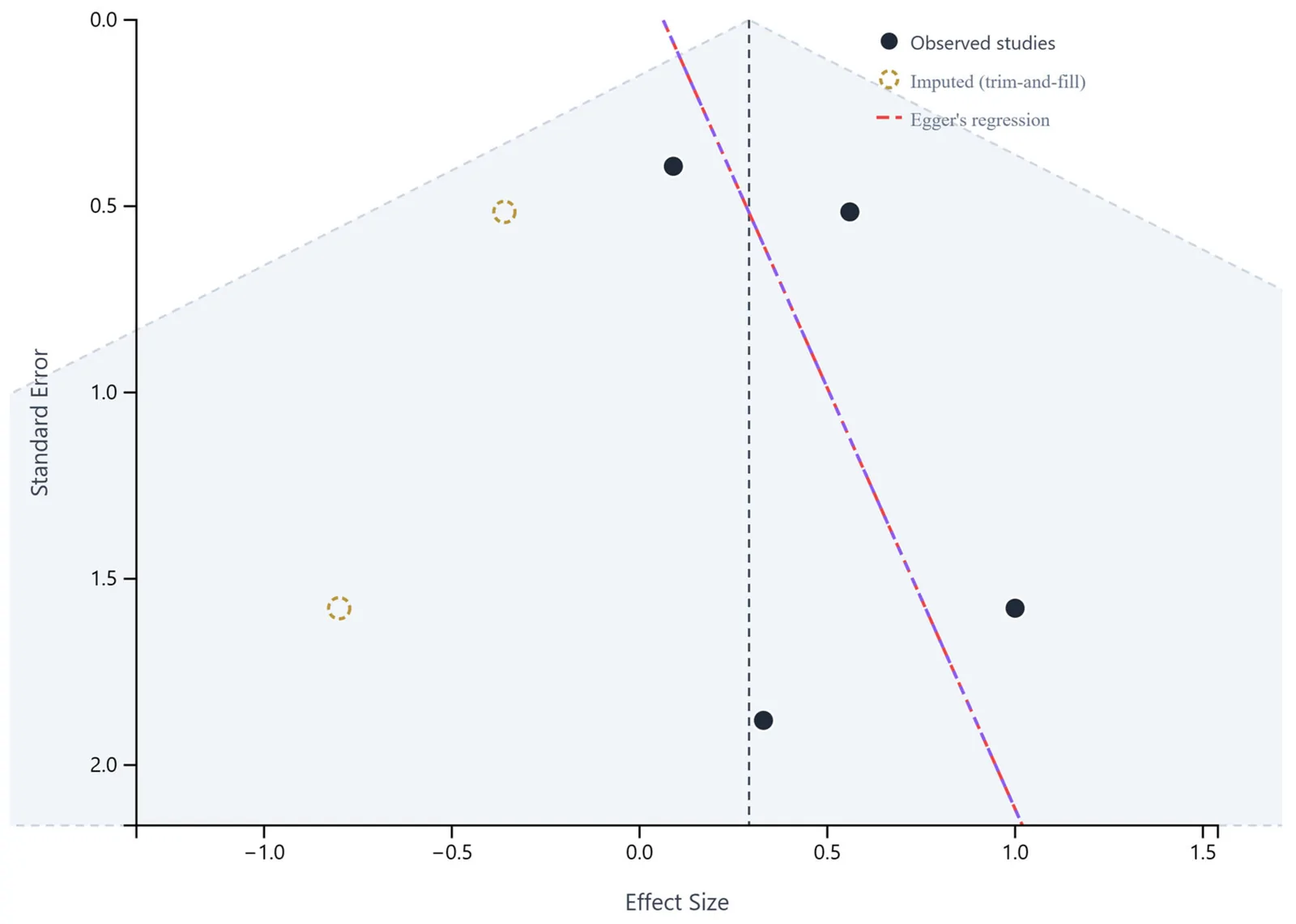
Funnel plot of included studies that report wound infection.

Publication bias was assessed using a funnel plot of four studies, supplemented by Egger’s regression test and Begg’s rank correlation test. Visual inspection of the funnel plot showed no substantial asymmetry, consistent with the results of Egger’s test (intercept = 0.44, t(2) = 0.90, *p* = 0.465) and Begg’s test (tau = 0.333, *p* = 0.734). Duval and Tweedie’s trim-and-fill procedure imputed two missing studies, yielding an adjusted pooled estimate of 0.100 (95% CI: −0.404 to 0.604), compared with the unadjusted estimate of 0.292. WAAP-WLS analysis was used as an additional bias-correction method. The WLS estimate was 0.063 (95% CI: −0.523 to 0.648). No studies met the adequacy threshold (SE ≤ 0.075) for 80% power to detect the WLS-adjusted effect, so the WLS estimate was used as a fallback (Figure 20).

**Figure 21:**
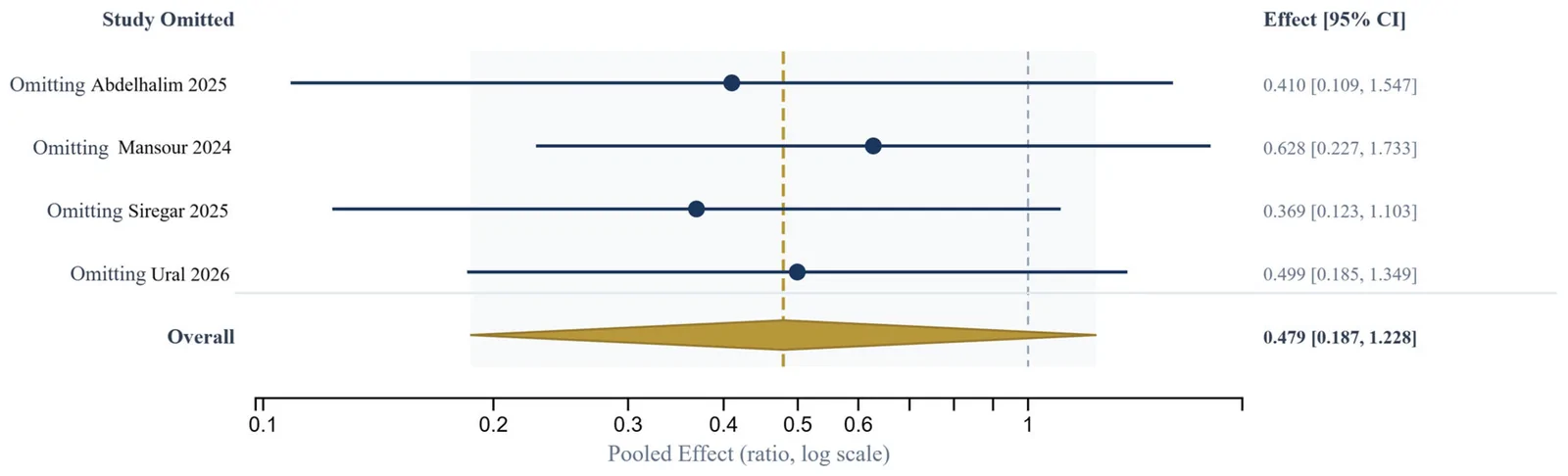
Leave-one-out sensitivity analysis plot of wound infection. Abdelhalim 2025 [44]; Mansour 2024 [46]; Siregar 2025 [50], Ural 2026 [47].

Leave-one-out sensitivity analysis was performed. No study exceeded the standard influence cutoffs. Mansour et al. had the largest influence, shifting the pooled estimate from 0.479 to 0.628 when removed. No single study removal altered the significance or direction of the pooled estimate (95% CI: 0.187 to 1.228), indicating robust results (Figure 21).

#### 3.5.7. Glans Dehiscence

Glans or wound dehiscence outcomes were provided by six of the included studies, generally revealing low overall rates of this complication. In the trials conducted by Abdelhalim et al., the incidence of glans dehiscence remained equivalent between groups (1 vs. 1 case—3.12% vs. 2.94%), a finding similar to Elnashar et al. (1 case per group—3.33%). Guinot et al. and Siregar et al. recorded no instances (0 cases) of glans dehiscence across any of their comparative cohorts. In contrast, Abdelazim et al. reported one event of glans dehiscence in their PRF arm (5%) compared to zero in the preputial/dartos flap arm. Wishahy et al. reported equivalent four vs. four wound-related dehiscence cases between groups since there was only fistula closure, hence no formal glansplasty (20% vs. 23.52%).

**Figure 22:**
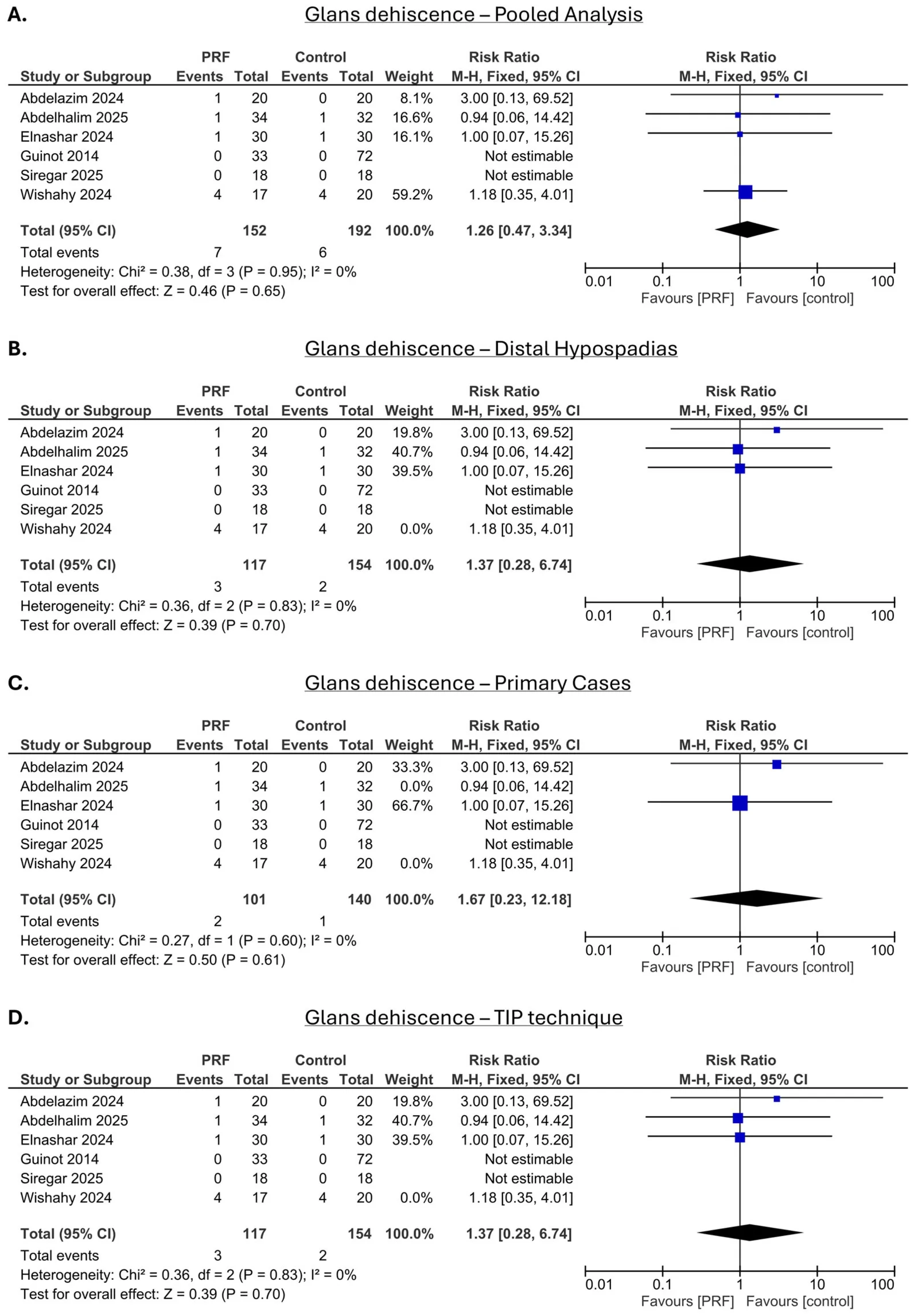
Forest plots of glans (or wound) dehiscence: (**A**) pooled effect analysis of all included studies, (**B**) subgroup analysis of distal hypospadias cases, (**C**) subgroup analysis of primary hypospadias cases, (**D**) subgroup analysis of TIP technique cases. Abdelazim 2024 [43]; Abdelhalim 2025 [44]; Elnashar 2024 [45]; Guinot [49]; Siregar 2025 [50], Wishahy 2024 [48].

The pooled RR was 1.26 (95% CI: 0.47 to 3.34), showing favorable outcomes for the control cases with no PRF membrane usage. Similar results indicating less glans dehiscence events for controls can be found in each subgroup analyzed, with wide CIs and no statistical significance. Heterogeneity was assessed as low using Chi-squared statistic (Chi^2^ = 0.38, df = 3, *p* = 0.95) and the I-squared statistic (I^2^ = 0%) (Figure 22).

**Figure 23:**
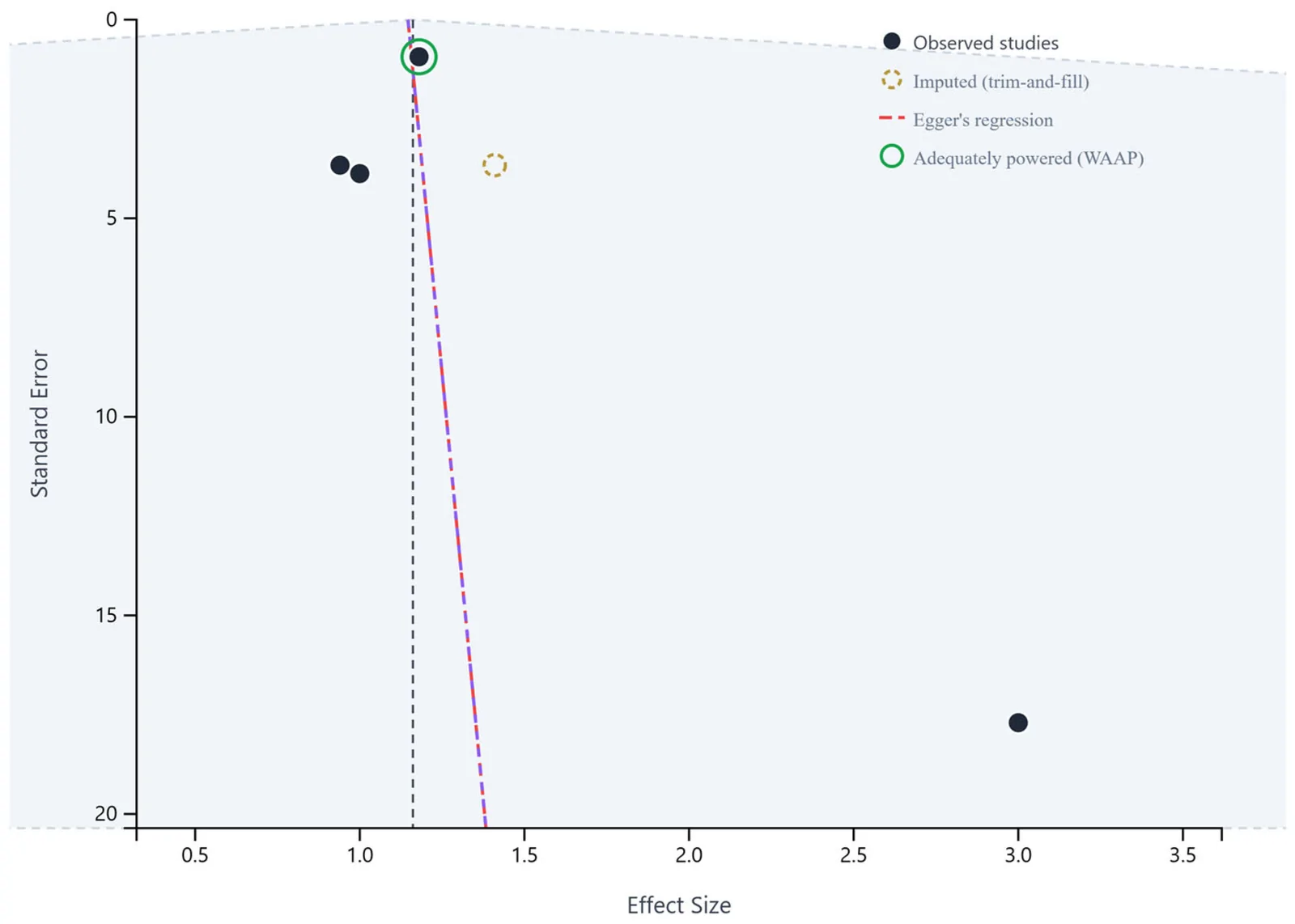
Funnel plot of included studies that report glans or wound dehiscence.

Publication bias was assessed using a funnel plot of four studies, supplemented by Egger’s regression test and Begg’s rank correlation test. Visual inspection of the funnel plot showed no substantial asymmetry, consistent with the results of Egger’s test (intercept = 0.01, t(2) = 0.18, *p* = 0.876) and Begg’s test (tau = 0.333, *p* = 0.734). Duval and Tweedie’s trim-and-fill procedure imputed one missing study, yielding an adjusted pooled estimate of 1.175 (95% CI: −0.502 to 2.852), compared with the unadjusted estimate of 1.161. WAAP-WLS analysis was used as an additional bias-correction method. The WLS estimate was 1.146 (95% CI: 0.919 to 1.374). One of four studies was adequately powered (SE ≤ 1.362) to detect the WLS-adjusted effect at 80% power. The WAAP estimate, based on the inverse-variance weighted average of these adequately powered studies, was 1.180 (95% CI: −0.650 to 3.010) (Figure 23).

**Figure 24:**
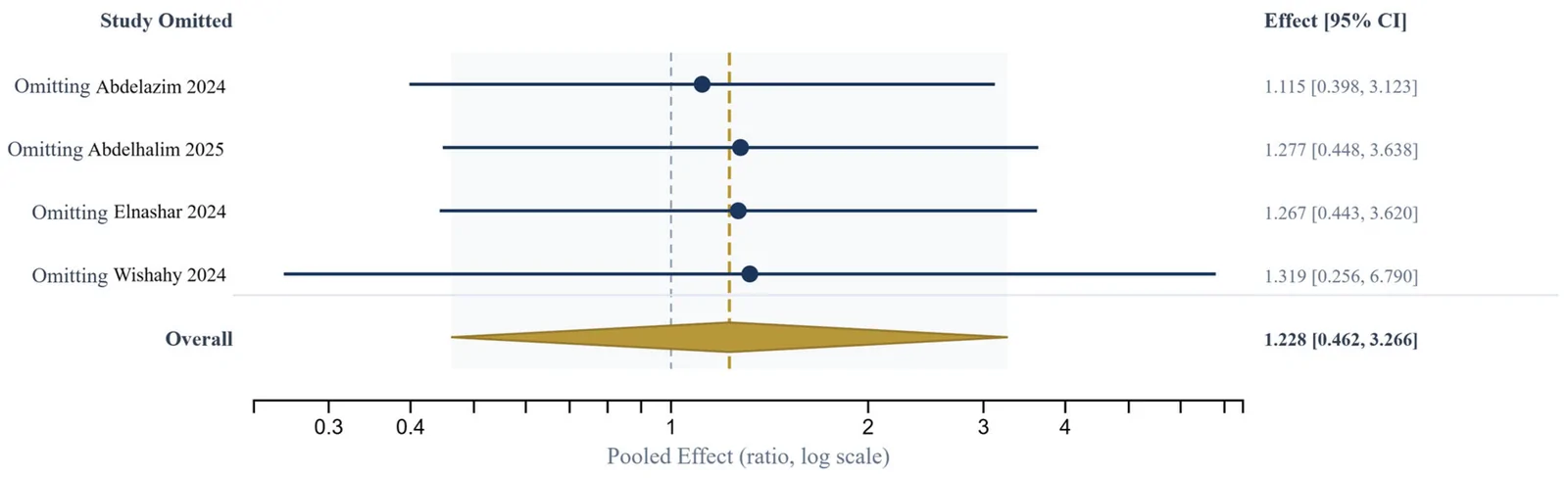
Leave-one-out sensitivity analysis plot of glans dehiscence. Abdelazim 2024 [43]; Abdelhalim 2025 [44]; Elnashar 2024 [45]; Wishahy 2024 [48].

No study exceeded the standard influence cutoffs during sensitivity analysis. Abdelazim et al. had the largest influence, shifting the pooled estimate from 1.228 to 1.115 when removed. No single study removal altered the significance or direction of the pooled estimate (95% CI: 0.462 to 3.266), indicating robust results (Figure 24).

#### 3.5.8. Bleeding or Hematoma Formation

Postoperative bleeding or hematoma formation was documented across three of the included trials. Abdelhalim et al. noted one event (3.12%) in their dartos flap layer arm and zero occurrences within their PRF arm. Siregar et al. reported two bleeding events occurring in the dartos flap group compared to one event in the PRF cohort (11.11% vs 5.55%). Elnashar et al. documented one hematoma event per study group (3.33%).

**Figure 25:**
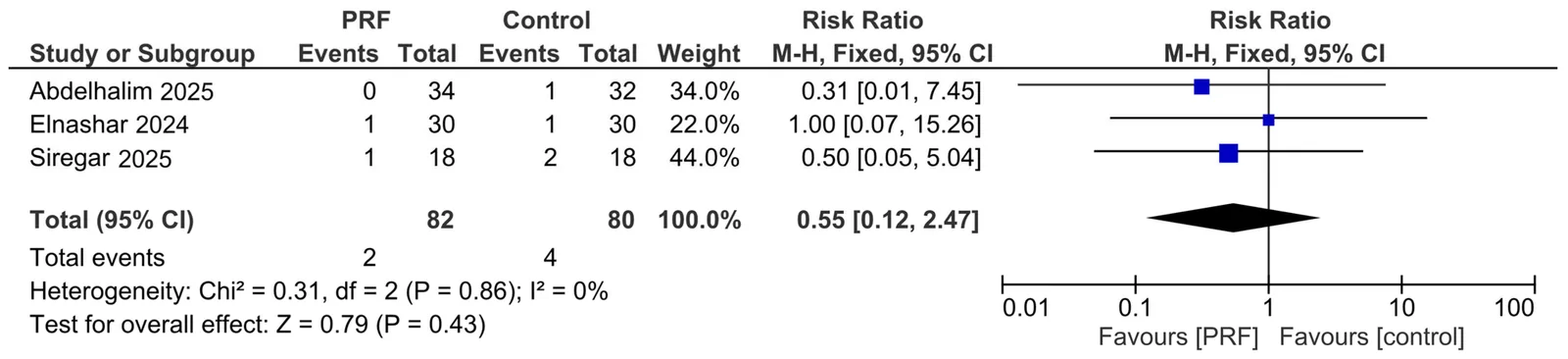
Forest plot of included studies that report bleeding events. Abdelhalim 2025 [44]; Elnashar 2024 [45]; Siregar 2025 [50].

A fixed-effect Mantel-Haenszel method was used to synthesize the results of eight studies. The pooled RR was 0.55 (95% CI: 0.12 to 2.46, *p* = 0.43), favoring the PRF group. However, the wide CI, the low number of events and studies reporting this outcome should lead to careful interpretation of results. Subgroup analysis was not conducted due to the low number of studies reporting this outcome. Heterogeneity was assessed as low using Chi-squared statistic (Chi^2^ = 0.31, df = 2, *p* = 0.86) and the I-squared statistic (I^2^ = 0%) (Figure 25).

**Figure 26:**
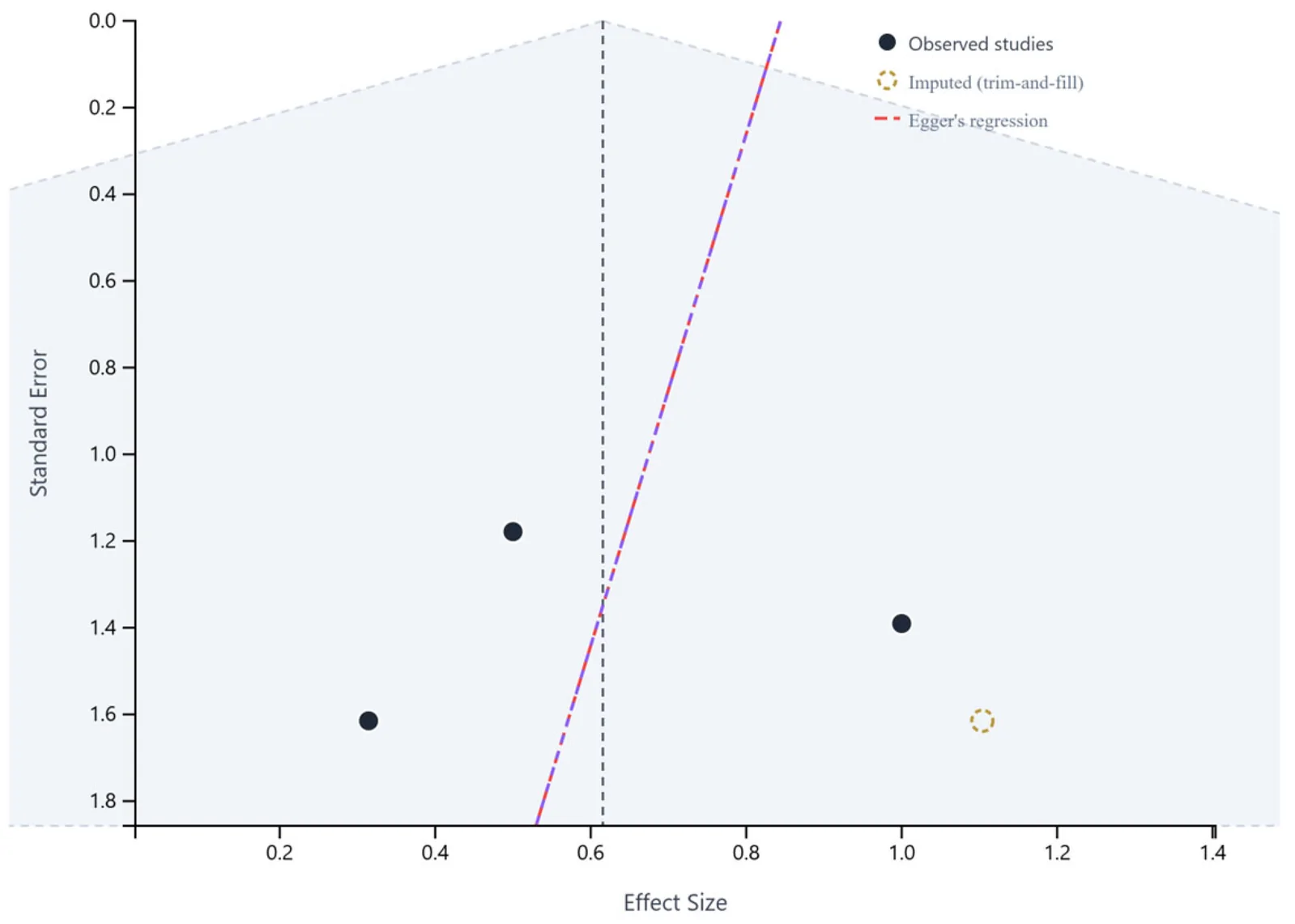
Funnel plot of included studies that report bleeding events.

Publication bias was assessed using a funnel plot of three studies, supplemented by Egger’s regression test and Begg’s rank correlation test. Visual inspection of the funnel plot showed no substantial asymmetry, consistent with the results of Egger’s test (intercept = −0.17, t(1) = −0.11, *p* = 0.931) and Begg’s test (tau = −0.333, *p* = 1.000). Duval and Tweedie’s trim-and-fill procedure imputed one missing study, yielding an adjusted pooled estimate of 0.709 (95% CI: −0.675 to 2.094), compared with the unadjusted estimate of 0.616. WAAP-WLS analysis was used as an additional bias-correction method. The WLS estimate was 0.844 (95% CI: −3.323 to 5.011). No studies met the adequacy threshold (SE ≤ 1.003) for 80% power to detect the WLS-adjusted effect, so the WLS estimate was used as a fallback (Figure 26).

#### 3.5.9. Certainty of Evidence

**Figure 27:**
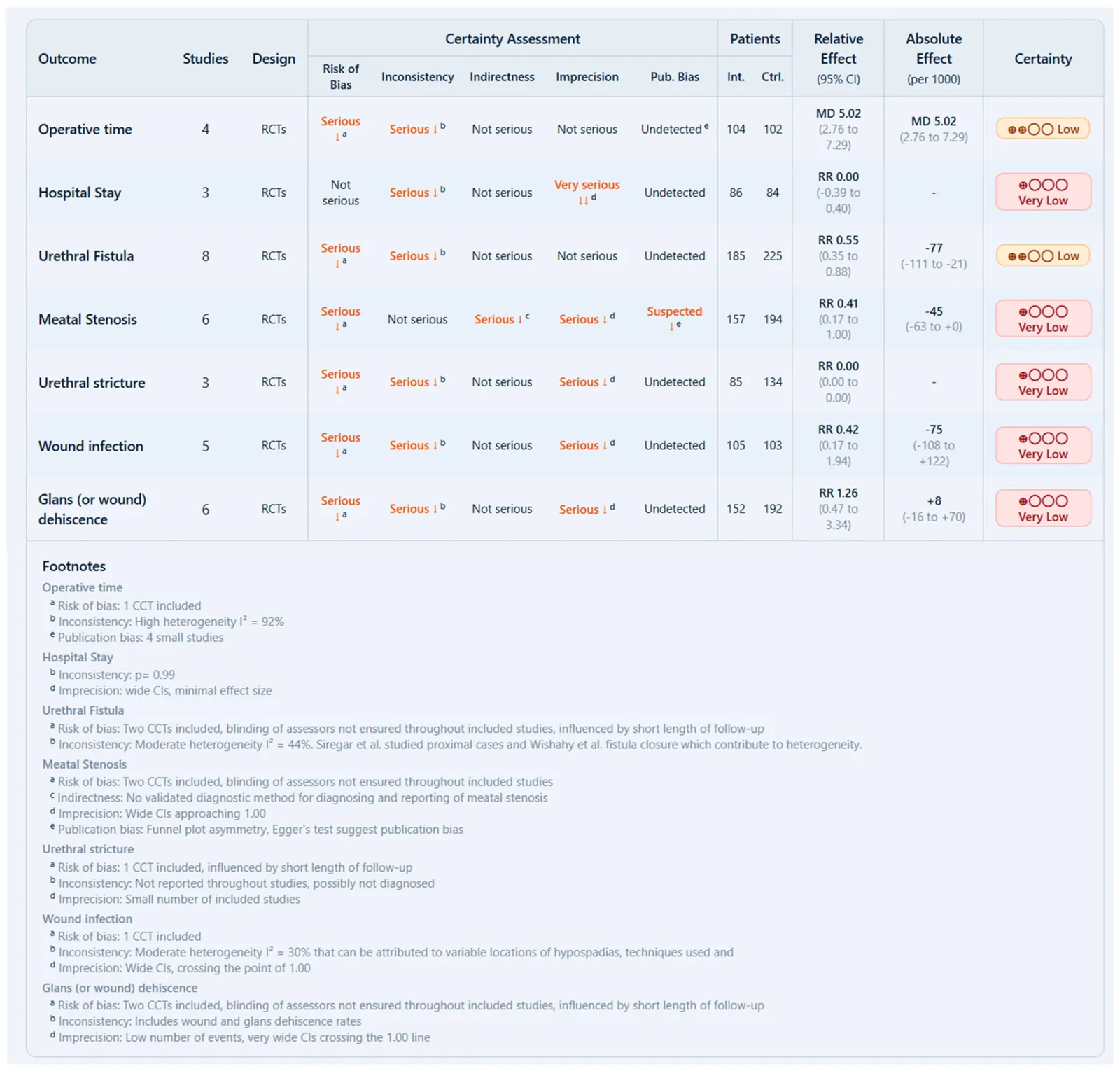
GRADE of evidence—Summary of Findings Table. CCT: Comparative Clinical Trial, CI: confidence interval, MD: mean difference, RCT: Randomized Clinical Trial, RR: Risk Ratio.

The aforementioned results have been compiled into a summary of findings table (Figure 27). This review included high-quality CCTs along with a small number of RCTs with a small number of patients. There may be some instances of risk of bias, inconsistency and imprecision, so certainty of evidence has been assessed as very low for most outcomes and low for operative time and urethral fistula. When considering subgroup analyses, evidence may provide a higher degree of certainty due to higher homogeneity of data and study designs. For example, for the urethral fistula RR, imprecision is lower when considering the distal hypospadias and TIP technique subgroups. Evidence was evaluated according to the GRADE framework using the GRADE handbook [39].

## 4. Discussion

This systematic review and meta-analysis of eight studies with application of PRF as an interposition coverage layer over a hypospadias urethroplasty, and in the case of Wishahy et al. a PRF coverage layer over a urethral fistula repair, provided the aforementioned results that may provide implications for practice and research.

PRF was introduced and pioneered by Choukroun et al. in 2001 and heralded as a second-generation autologous platelet-derived product, with no anticoagulants needed and with higher concentrations of leukocytes and associated growth factors [51]. This is in contrast to fibrin glue or sealants that are not autologous and can be costly to produce. Additionally, PRF is produced more readily versus the first-generation product of PRP, which needs more laborious preparation. PRP requires two centrifugation cycles, addition of bovine thrombin and anticoagulants, it must be administered preferably within 10 min of production and it may rarely lead to allergic reactions or coagulopathies [62]. PRF presents clinical advantages over PRP such as the fact that it is fully autologous, thus eliminating any immune reaction potential side effects, without needing any biochemical processing or additions. The PRF matrix contains various growth factors, leukocytes and cytokines that promote tissue healing, demonstrating prolonged release relative to PRP. Furthermore, PRF production remains cost-effective and easy, requiring only a single centrifugation step and no additives with the PRF membrane showing high flexibility and elasticity. This makes PRF production feasible in the operating room where the application and fixation of the PRF membrane can be completed immediately after its production, as seen in many included studies. This process bypasses the drawbacks of PRF usage because the blood drawing and handling should be expedient since no anticoagulants are used and the membrane structure can modulate over time. Several protocols have been employed over the past years, with modifications in centrifugation speed and time or in blood container materials replacing glass or plastic with titanium, thereby providing different PRF products such as Advanced-PRF or Titanium-PRF, respectively [34].

A multitude of PRF protocols have been used across the included studies, so it stands to reason that PRF production methods can influence the analyzed clinical outcomes. A significant notice should be given that even naming in the field is variable and inconsistent Specifically, a study excluded from this review by Eryilmaz et al. used PRP gel that closely resembles PRF, but the addition of calcium chlorate precludes its categorization as such [55]. In contrast, Abdelhalim et al. and Mansour et al. describe the PRF membrane manufacturing process according to the original process by Choukroun et al. [44,46,51]. Similarly, all included studies provide highly variable centrifugation speeds and durations, so heterogeneity is high in this regard. RCF or g-force is not uniformly reported and it should provide more objective information than RPM. Further research in animal models should guide the further development of PRF. As an example, Soyer et al. studied the effects of PRF on growth-factor levels in urethral repair in an animal model of Wistar albino rats and they found increased levels of TGF-β-R and VEGF [63]. The same authors also published a preliminary report on PRF application for urethrocutaneous fistula repair in a three-year old child [64]. This translational research is essential in finding the most appropriate PRF formulation and further standardizing it. This could lead to increased adoption of PRF and better homogeneity among studies.

Regarding the results of this review, operative time was slightly prolonged by 5.02 min (95% CI: 2.76 to 7.29) when a PRF membrane was applied, a statistically significant finding (*p* < 0.0001) for both pooled and most subgroup analyses. The subgroup of cases that applied a PRF membrane as a solitary layer did not lead to prolonged operative time (*p* = 0.38). PRF must be produced during surgery and is a novel technique [34,65]. It should be noted that 5 min does not substantially prolong a reconstructive hypospadias operation. The use of PRF is novel and therefore parameters such as learning curve, surgical and operating room team factors should be considered when interpreting these results. Variability among individual surgeons and centers may certainly influence the heterogeneity found for this outcome. Familiarization with PRF production, parallelization of tasks during surgery and inclusion in routine use by operative teams may lead to reductions in operative time. As with all surgical techniques, learning curves must be overcome to fully appreciate the results [65,66].

The length of hospital stay was mostly similar, a finding that may be explained as hospital stay in clinical practice is usually dictated and affected by surgeon and parent preferences, hospital cost variability and health system policy factors. Differences in hospital stay could also be influenced by postoperative complications and their treatment. Enhanced recovery after surgery (ERAS) protocols are also being adopted worldwide that aim to minimize hospital stay [67]. The coverage layer choice is a minor factor that is not likely to affect hospital stay in routine clinical practice.

Urethral fistula appearance rate is a common and important complication that usually needs to be corrected by another operation, although non-operative management has been reported [21,68]. Thus, all eight included studies reported this outcome. Wishahy et al. reported recurrence of corrected fistulas. Across the eight studies, PRF layer application appears to lower urethral fistula rates with a statistically significant RR of 0.55 (95% CI: 0.35 to 0.88), even when used as a solitary layer (RR = 0.62–95% CI: 0.38–0.99, *p* = 0.05). Similar results are noted among subgroups, mainly for distal hypospadias and TIP subgroups with statistical significance and narrower CIs. Risk of bias in the included studies leading to underreporting of urethral fistula occurrence may arise due to the limited follow-up time in many studies, as well as selection bias and publication bias. This limits the certainty of evidence to low. A very important aspect of included studies is the limited length of follow-up. Late occurrence of urethral fistula has been documented, even years after the primary operation [69]. Conversely, spontaneous healing of urethral fistula has been recorded [43,70]. Future studies should aim for at least a year of follow-up to provide more certainty in recorded outcomes of fistula occurrence.

The quest for the optimal urethroplasty technique is never-ending. Even for widely adopted techniques, the choice of coverage layer over the urethroplasty may be difficult. Recognizing and mitigating risk factors for urethral fistula formation is likewise challenging [71]. One, two or no waterproofing protective layers remain valid choices under active clinical application and research. Options include local ventral or dorsal dartos flap, tunica vaginalis, de-epithelized skin, spongiosum and recently PRF [25,28]. There are reports supporting the use of double coverage layers for TIP urethroplasty, claiming reduced complication rates of urethral fistula <5% [72,73]. A study by Zulli et al. opposes this concept, maintaining that a well-performed urethroplasty suture line is the main predictor of urethral fistula appearance rather than a second layer [74]. A layer of PRF membrane, less than 1 mm thick, appears to stand in the middle ground of this debate since it can operate both as a physical barrier and as a healing factor matrix, without the need for further tissue handling and the risks related to acquisition and insertion of a second layer.

Meatal stenosis remains an important consideration during the postoperative course of hypospadias, as it represents a type of urethral stricture that may lead to urethral fistula or diverticulum formation [75,76]. The RR of all studies was 0.41 (95% CI: 0.17 to 1.00, *p* = 0.05), suggesting a statistically significant reduction in meatal stenosis for the PRF group. This was enhanced by subgroup analysis with statistically significant reduction for distal cases (RR = 0.33–95% CI: 0.12–0.87, *p* = 0.03) and single layer subgroups (R = 0.33–95% CI: 0.12–0.92, *p* = 0.04) with upper CIs further away from the 1.00 point. In contrast, the primary case subgroup showed a higher RR and wider CIs crossing the RR point of 1.00, meaning imprecision, that could account for the pooled effect estimate CI width. Close follow-up and prompt evaluation for potential meatoplasty are essential for improved outcomes [77,78]. Clinical research should also document the method of meatal stenosis diagnosis like catheterization or caliber of the urinary stream, and whether calibration was employed postoperatively. Grading systems have been proposed but without universal adoption [79].

Stricture of the neourethra was found to be not estimable with three studies reporting zero strictures and the rest not reporting this outcome. However, urethral stricture could have been evaluated by methods such as inspection of the penis or the urinary stream without formal catheterization, so its method of evaluation should be noted [80]. Cases of stricture after hypospadias repair have been diagnosed even in adulthood, so follow-up should be extended to document this outcome in future research [81].

Wound infection RR was 0.42, favoring the use of PRF, approaching but not reaching statistical significance. Confidence in this pooled effect must be cautious since the upper CI crosses the boundary of 1.00 (95% CI: 0.17 to 1.04, *p* = 0.06). Only the TIP technique subgroup showed statistically significantly fewer wound infections with a RR of 0.32 (95% CI: 0.11–0.96, *p* = 0.04). Two of the three studies in this subgroup used PRF as an adjunctive layer. In contrast, single PRF layer subgroup analysis presented a RR of 0.69 with wide CIs, suggesting careful consideration of this effect. The suggested advantage of a PRF membrane layer could be the result of the composition of the PRF matrix with healing factors, growth factors, leukocytes, cytokines, fibrin and many more that enhance natural wound healing and provide antimicrobial resistance [82]. There is a degree of risk of bias when assessing this outcome due to confounding factors such as antibiotic selection, mode of administration, catheter type, type of local antiseptic used or bandage selection among others. These also contribute to the moderate heterogeneity observed. Studies do not always report the scheme of perioperative or postoperative antibiotics. In contrast, some researchers have questioned the use of prophylactic preoperative antibiotics for hypospadias surgery [83].

Dehiscence of the glans is a complication with low frequency of events across the included studies but with an advantage given to the control group. The meta-analysis yielded a non-statistically important RR of 1.26 (95% CI: 0.47 to 3.34), showing lower incidence of glans or wound dehiscence for the control cases with no PRF membrane usage. This result was echoed during the subgroup analyses. Two of the included studies reported no glans dehiscence [49,50]. Overall incidence of dehiscence was low among groups. As glansplasty requires low or no tension over the suture line, consideration should be given to the bulkiness of the underlying layers, even if anatomical parameters have been suggested to influence frequency of this outcome, like glans width < 15 mm and urethral plate width < 8 mm [84]. Other factors implicated in increasing the incidence of glans dehiscence are proximal or re-do hypospadias repairs, caudal block anesthesia use, surgery performed on infants less than 6 months old or surgery after puberty [84].

Bleeding or hematoma formation was also quite similar in the studies where it was reported, with Abdelhalim et al. and Siregar et al. reporting bleeding in one control (3.12%) vs. 0 PRF cases and two controls vs. one PRF case (11.11% vs. 5.55%) respectively. Elnashar et al. documented a single bleeding event in each group. The pooled RR of 0.55 (95% CI: 0.12 to 2.47, *p* = 0.43) shows an advantage for the PRF layer application but the wide CI, the low number of events and studies reporting this outcome lead to limited confidence in the results. Bleeding and hematoma formation confounders can be attributed to technical operative factors, bipolar cautery use, tourniquet use, infiltration with vasoactive medications and compression bandage selection [19,85,86]. Early timing of dorsal dartos flap preparation has been suggested to reduce bleeding in recent research [87]. PRF in principle should display the hemostatic sealant effect of fibrin glue [88].

Several previous reviews have investigated this topic with a different focus. The review by Moran et al. included studies only pertaining to primary hypospadias treated in a single-stage with platelet-derived products or with acellular matrix or tissue adhesives, displaying reduction in urethral fistula frequency in the groups that used these products [89]. A 2023 review by Borkar et al. analyzed four RCTs involving 355 patients. They found that autologous platelet concentrates (APC) lowered the rates of wound infections, meatal stenosis, and overall complications. However, there was no notable difference between groups regarding urethral fistula rates [90]. Xiao et al. performed a systematic review and meta-analysis of studies describing the use of PRF platelet-rich fibrin or APC products for the coverage of primary hypospadias corrected by TIP urethroplasty. They documented statistically significantly lower rates of urethral fistula, wound infection, meatal stenosis and total complications, in support of APCs as an additional layer [91]. Accordingly, this review demonstrates statistically significant reductions in urethral fistula rates, meatal stenosis incidence and wound infection rates (for the TIP subgroup) but also prolonged operative time for the PRF cases. In contrast, higher rates of glans dehiscence are shown in this study but without statistical significance. Finally, the retraction of the study by Mahmoud et al., leads to careful appreciation of the results of previous reviews, since its results have been questioned after the article’s retraction [92].

When considering the implications for clinical practice, our findings reinforce the safety of PRF membrane layers and the reproducibility of the PRF membrane production process. When no other layer or flap is available, evidence suggests that PRF membrane is a valid alternative to reinforce the urethroplasty and may protect from complications. Although the certainty of evidence is low, PRF membrane layers may likely lower urethral fistula occurrence, wound infection and meatal stenosis in selected cases like distal or TIP cases versus dartos flaps. Surgeons should utilize the techniques most familiar to them, until further research is conducted.

Although these results seem encouraging, many factors affecting the aforementioned outcomes must be reported as they limit the interpretation of the results of this study. These include surgical factors, PRF formulation and production factors, methods of reporting results, publishing bias, number of included participants and many more. For example, ischemia and its duration have been studied [93]. Similarly, suture material and continuous or not sutures remain topics of debate for hypospadias surgery [94]. Confounding of perioperative factors and causality between surgical parameters and outcomes need further strictly designed and high-volume research to distinguish [95]. All these factors should be further controlled and standardized, at least when reported in the literature, to promote healthy research to reach potential conclusions to inform hypospadias surgeons. Clinical researchers may opt to objectively report these operative parameters in the future.

Other limitations of this study include that this review was not registered. Publication bias of some included studies may be inferred due to the limited follow-up duration. The variability in patient age between studies may influence outcomes. The inclusion of a study of fistula closure may be a source of heterogeneity since it is not a hypospadias reconstructive operation, although this is debatable for cases that present with multiple fistulas in various locations. Another limitation could arise due to the geographic concentration of most selected studies within a single country. Patient groups in included studies may be small in number and thus may be underpowered to provide certainty in the results. Finally, some studies report on multiple locations of hypospadias and primary or redo cases together, without presenting separate results for relevant subgroups. Conflating the results may produce heterogeneity and imprecision.

Further large-scale studies are warranted to provide more robust evidence. More multicenter trials are needed to mitigate variability in surgical factors. As some postoperative complication outcomes are rare, larger cohorts are essential to strengthen the certainty of evidence. The subgroup using PRF as an additional layer was small and therefore this is a valid topic of interest that can be explored by future studies. Longer follow-up periods are very important and essential to provide long-term results, ideally until puberty or even adulthood. Future studies should include more severe hypospadias cases as most are distal in the present literature. Standardized reporting of PRF production and of relevant outcomes should be upheld so that future research can be of high quality. A remark on blinding of outcome assessors should be made. Blinding was not commonly reported, therefore probably not implemented, and its implementation can strengthen clinical trials.

## 5. Conclusions

The integration of personalized autologous biological derivatives, specifically a PRF membrane, as an intermediate coverage layer in hypospadias repair may offer potential clinical advantages. The incorporation of these growth-factor-rich matrices may lower the incidence of various postoperative complications.

Theoretically, it could be argued that PRF membrane layer as an additional secondary layer to cover the urethroplasty could be a safe, effective, and reproducible surgical strategy to reduce morbidity in patients undergoing hypospadias repair, but evidence is currently lacking on the matter. Only two studies reported data on adjunctive PRF layer usage. However, the present evidence does suggest that PRF membrane layers are likely to reduce the incidence of urethral fistula, meatal stenosis and wound infection with slightly longer operative times but may also lead to higher glans dehiscence rates vs. dartos flap use, for selected subgroups of patients. As a personalized autologous graft, PRF membrane can become a valid choice for hypospadias surgeons when other layers are unavailable or difficult to acquire. However, heterogeneity in PRF preparation, surgical technique, comparator layers, patient selection, and follow-up precludes firm conclusions regarding superiority or long-term safety. Certainty of evidence is mainly low. Data for outcomes such as length of hospital stay, urethral stricture, and hematoma formation were inconsistently captured across the included trials, limiting broader statistical synthesis. Standardized, adequately powered randomized trials with uniform outcome definitions and longer follow-up are required. Uniform clinical protocols to address the aforementioned shortcomings, with long-term follow-up timelines, are necessary. After research and standardization, these protocols should be used in the future to fully capture delayed stenosis, stricture, fistula recurrences or other outcomes.

## Figures and Tables

**Table 1:** Publication details of studies selected for inclusion.

	Author	Type of Study	Study Period	Surgeons and Institutions Involved	Year of Publication	Follow-Up Length (Months)	Country	Reference
1	Abdelazim et al.	Single-center prospective randomized controlled trial (RCT)	April 2017–February 2018	Single surgeon/Cairo University Specialized Pediatric Hospital	2024	6 (range 2–10)	Egypt	[43]
2	Abdelhalim et al.	Single-center prospective RCT	n/a (possibly 2022–2025)	Single surgeon/Suez Canal University Hospital	2025	6–12	Egypt	[44]
3	Elnashar et al.	Single-center prospective RCT	March 2021–March 2023	Four surgeons/Mansoura University Children Hospital	2024	9 (range, 5–17)	Egypt	[45]
4	Guinot et al.	Single-center prospective non-randomized comparative study with historical control group	June 2010–September 2011 (historical control group 2008–2009)	Two surgeons/University Hospital of Rennes	2014	8 (range, 6–18)	France	[49]
5	Mansour et al.	Single-center prospective RCT	September 2019–March 2022	Single surgeon/Suez Canal University Hospital	2024	17.9 (range, 6–30)	Egypt	[46]
6	Siregar et al.	Single-center prospective non-randomized comparative study	September 2023–December 2024	Two surgeons/Hasan Sadikin general hospital	2025	6	Indonesia	[50]
7	Ural et al.	Single-center prospective RCT	January 2023–March 2024	Single Surgeon/Kahramanmaras Sutcu Imam University	2026	3	Turkey	[47]
8	Wishahy et al. (fistula)	Single-center prospective RCT	February 2021–February 2022	Multiple surgeons/Cairo University	2024	3 (minimum)	Egypt	[48]

RCT: Randomized Controlled Trial.

**Table 2:** Details of included studies’ patient characteristics.

	Author	Location of Urethral Meatus	Surgical Technique/Urethroplasty	Protective (Coverage) Layer Applied per Group	Recruited Patients/Group	Age (Months)	Primary Operation or Reoperation	PRF as Single or Adjunctive Layer in Study Group	Reference
1	Abdelazim et al.	Distal	Tubularized Incised Plate (TIP)	Preputial or dartos flap	20	27.1 ± 17.7	Primary	Single	[43]
				Platelet-rich fibrin (PRF)	20	24.8 ± 19.2			
2	Abdelhalim et al.	Distal	TIP	Dartos flap layer	32	47.78 (SD 24.548)	Second operation (failed primary)	Single	[44]
				PRF	34	43.76 (SD 18.961)			
3	Elnashar et al.	Distal	TIP	PRF	30	18.5 (range 13–30.75)	Primary	Single	[45]
				Ventral dartos flap	30	17 (range 11–27.3)			
4	Guinot et al.	Distal	TIP	PRF	33	8 (range 3.5–67)	Primary	Single	[49]
				Dorsal foreskin subcutaneous flap	72				
5	Mansour et al.	Distal	TIP	Pedicled dartos flap with PRF	22	30 ± 21	Primary	Adjunctive	[46]
				Pedicled dartos flap	22	38 ± 28			
6	Siregar et al.	Proximal	Two-stage Thiersch-Duplay	Dartos flap	18	9.7 ± 5.7	Primary	Single	[50]
				Bio-PRF	18	7.3 ± 3.2			
7	Ural et al.	Distal, mid-penile	TIP	Ventral dartos flap + PRF	11	31 ± 12	Primary	Adjunctive	[47]
				Ventral dartos flap	11	30 ± 11			
8	Wishahy et al. (fistula)	Urethral Fistula—Various locations	Fistula closure	Dartos flap	20	20.6 (range 6–45)	Second operation (failed primary with urethral fistula)	Single	[48]
				PRF	17	22.45 (range 11–56)			

PRF: platelet-rich fibrin, SD: standard deviation, TIP: Tubularized Incised Plate.

**Table 3:** Details of included studies’ PRF Membrane Formulations.

	Author	Name Used to Refer to the Product	Centrifugation Characteristics	Use of Anticoagulant	Use of Pro-Coagulant	Converted to Thin Membrane	Coverage Layer Fixation Technique (Quote from Study)	Volume of Drawn Blood and Container	Classification as PRF and Deviation from Commonly Used Protocol	Reference
1	Abdelazim et al.	PRF	1 cycle 3500 RPM 20 min	No	No	Yes	“Overlying the PRF extract directly over the repair and fixation in the four corners by Vicryl 6-0 sutures”	5 mL in plastic tube with no additives	Yes, minor deviation in RPM and prolonged time	[43]
2	Abdelhalim et al.	PRF	1 cycle 2700 RPM 12 min	No	No	Yes	“An autologous PRF membrane was used and secured by polygalactin 6/0”	10 mL in glass or glass-coated plastic tube	Yes, Choukroun’s PRF–L-PRF	[44]
3	Elnashar et al.	PRF	1 cycle 3000 RPM 12 min	No	No	Yes	“After urethroplasty, the PRF sheet was anchored over the neourethra with absorbable polyglyconate 6/0 interrupted sutures”	5–10 mL in standard plain sterile glass tube	Yes, slightly increased RPM	[45]
4	Guinot et al.	PRF membrane	1 cycle 3000 RPM (400× *g* RCF) 10 min	No	No	Yes	“This autologous PRF membrane was applied over the urethroplasty and secured using a few resorbable polyglyconate 7/0 interrupted sutures”	5–10 mL in sterile plain glass tube	Yes, minor deviation in RPM (but not RCF) and reduced time	[49]
5	Mansour et al.	PRF membrane	1 cycle 2700 RPM (400× *g* RCF) 12 min	No	No	Yes	“the PRF membrane was secured upon urethroplasty and secured with 6/0 polyglactin few interrupted sutures”	10 mL in sterile glass tube without anticoagulant	Yes, Choukroun’s PRF–L-PRF	[46]
6	Siregar et al.	Bio-PRF gel layer, Bio-PRF membrane	1 cycle 7× *g* RCF 8 min	No	No	Yes	“Sutured to the surrounding tissue using 6-0 polydioxanone”	10 mL in plain collection tube without anticoagulant	Yes, reduced RCF and time	[50]
7	Ural et al.	PRF membrane	1 cycle 2800 RPM 12 min	No	No	Yes	“The membrane was wrapped around the neourethra and fixed with four interrupted 8-0 polyglycolic acid sutures”	4.5 mL in citrate-free siliconized tubes	Yes	[47]
8	Wishahy et al. (fistula)	PRF membrane	1 cycle 3500 RPM 20 min	No	No	Yes	“overlying the PRF extract directly over the repair and fixation in the four corners by Vicryl 6-0 sutures”	5 mL in sterilized plastic tube with no additives	Yes, slightly increased RPM and time	[48]

PRF: platelet-rich fibrin, RCF: Relative Centrifugal Force, RPM: Rotations Per Minute.

**Table 4:** Details of included studies’ patient outcomes.

	Author	Protective (Coverage) Layer Applied	Recruited Patients/Group	Operative Time (min)	Length of Hospital Stay (Days)	Urethral Fistula	Meatal Stenosis	Urethral Stricture	Wound Infection	Glans Dehiscence	Bleeding or Hematoma	Reference
1	Abdelazim et al.	Preputial or dartos flap	20	N/A	N/A	7 (35%)	2 (10%)	N/A	0	0	N/A	[43]
		PRF	20	N/A	N/A	0 ^‡^	1 (5%)	N/A	0	1 (5%)	N/A	
2	Abdelhalim et al.	Dartos flap layer	32	102.8 (SD 9.972)	4.81 (SD 1.575)	6 (18.75% *)	4 (12.5%)	N/A	5 (15.62%)	1 (3.12%)	1 (3.12%)	[44]
		PRF	34	107.9 (SD 9.721)	4.82 (SD 1.678)	1 (2.94%)	1 (2.94%)	N/A	3 (8.82%)	1 (2.94%)	0	
3	Elnashar et al.	PRF	30	95.7 ± 11.9	5.47 ± 1.11	4 (13.33%)	1 (3.33%)	0	N/A	1 (wound) (3.33%)	1 (3.33%)	[45]
		Ventral dartos flap	30	102.3 ± 10.3	5.47 ± 0.89	3 (10%)	7 (23.33%)	0	N/A	1 (wound) (3.33%)	1 (3.33%)	
4	Guinot et al.	PRF	33	N/A	N/A	2 (6.06%)	0	0	N/A	0	N/A	[49]
		Dorsal foreskin subcutaneous flap	72	N/A	N/A	3 (4.16%)	0	0	N/A	0	N/A	
5	Mansour et al.	Pedicled dartos flap with PRF	22	105 ± 5	4 ± 1.68	1 (4.54%)	2 (9.09%)	0	0	0 (wound)	N/A	[46]
		Pedicled dartos flap	22	90 ± 9	4 ± 1.73	4 (18.18%)	2 (9.09%)	0	5 (22.72%)	0 (wound)	N/A	
6	Siregar et al.	Dartos flap	18	148.67 ± 6.32	N/A	9 (50%)	0	N/A	2 (11.11%)	0	2 (11.11%)	[50]
		Bio-PRF	18	151 ± 5.64	N/A	10 (55.55%)	1 (5.55%)	N/A	2 (11.11%)	0	1 (5.55%)	
7	Ural et al.	Ventral dartos flap + PRF	11	N/A ^†^	N/A	0	N/A	N/A	0	N/A	N/A	[47]
		Ventral dartos flap	11	N/A	N/A	2 (18.18%)	N/A	N/A	1 (9.09%)	N/A	N/A	
8	Wishahy et al.	Dartos flap	20	N/A	N/A	6 (30%)	N/A	N/A	N/A	4 (wound) (20%)	N/A	[48]
		PRF	17	N/A	N/A	2 (11.76%)	N/A	N/A	N/A	4 (wound) (23.52%)	N/A	

* Percentages reported until the second decimal point. ^†^ PRF preparation reported to have required less than 3 min, with no clinically meaningful prolonged operative time ^‡^ 2 (10%) reported urethral fistulas that after 6 months of follow-up closed spontaneously hence 0 reported fistula at endpoint of study. N/A: Not Available, PRF: platelet-rich fibrin, SD: standard deviation.

## Data Availability

The original contributions presented in this study are included in the article/Supplementary Materials. Further inquiries can be directed to the corresponding author.

## Supplementary Materials

The following supporting information can be downloaded at: https://www.mdpi.com/article/10.3390/jpm16100527/s1, Table S1: PRISMA 2020 Completed Checklist; Table S2: Details of included and excluded studies’ Platelet Rich Concentrate Membrane Formulations.

## Abbreviations

The following abbreviations are used in this manuscript:

CCT	Comparative Clinical Trial
N/A	Not Available
PRF	Platelet-rich fibrin
PRP	Platelet-Rich Plasma
RCT	Randomized Control Trial
RR	Risk Ratio
SD	Standard deviation
TIP	Tubularized Incised Plate

## Appendix A

### Appendix A.1

PubMed Search Strategy:

(“platelet-rich fibrin”[MeSH Terms] OR (“platelet rich”[All Fields] AND “fibrin”[All Fields]) OR “platelet rich fibrin”[All Fields] OR (“platelet”[All Fields] AND “rich”[All Fields] AND “fibrin”[All Fields]) OR “platelet-rich fibrin”[All Fields]) AND (“hypospadias”[MeSH Terms] OR “hypospadias”[All Fields] OR “hypospadia”[All Fields]).

### Appendix A.2

Scopus Search Terms:

TITLE-ABS-KEY(platelet rich hypospadias).
