# Supplementary material for: Platelet-Rich Fibrin Membrane as a Personalized Autologous Graft Layer for Hypospadias Surgery: Systematic Review and Meta-Analysis
Source: J Pers Med. 2026 Oct 12;16(10):527. doi: 10.3390/jpm16100527 (PMC13705990; doi:10.3390/jpm16100527)
Supplement: Supplementary file 1 [file jpm-16-00527-s001.zip › Supplementary Table S2 - Details of included and excluded studies’ Platelet Rich Concentrate Membrane Formulations.pdf]

Table S2. Details of included and excluded studies' Platelet Rich Concentrate Membrane Formulations.

Author	Name used to refer to the product	Centrifugation characteristics	Use of anticoagulant	Use of pro-coagulant	Converted to membrane	Coverage Layer Fixation technique	Volume of drawn blood and container	Classified as PRF and reasons for exclusion	Reference
1 Abdelazim et al.	*PRF*	1 cycle<br>3500rpm<br>20 minutes	No	No	Yes	"Overlying the PRF extract directly over the repair and fixation in the four corners by Vicryl 6–0 sutures"	5ml in plastic tube with no additives	Yes, minor deviation in rpm and prolonged time	[43]
2 Abdelhalim et al.	*PRF*	1 cycle<br>2700 rpm<br>12 minutes	No	No	Yes	"An autologous PRF membrane was used and secured by polygalactin 6/0"	10 ml in glass or glass-coated plastic tube	Yes, Choukroun's PRF – L-PRF	[44]
3 Dudhani et al.	*PRP gel*	2 cycles, "1st low spin cycle at 150G (soft spin cycle at 1400) for 5 min", second "(high spin cycle at 3400)"	Yes	Yes (autologous thrombin and calcium gluconate)	Not mentioned explicitly	"The platelet gel was secured using 6-0 Polyglactin 910 interrupted sutures"	9 ml for PRP production in EDTA vial and 3ml to prepare thrombin in plain vial	No, 2 cycles and additives used	[52]
4 Elghamry et al.	*PRP graft*	2 cycles<br>3000 rpm<br>10 minutes	No	Yes (calcium gluconate)	Yes	"held in place temporarily by gentle compression between two sterile surgical swabs for approximately 1—2 min, allowing it to adhere to the tissue surface. Then sutured using 7—0 interrupted polyglactin few stitches"	10 ml in sterile tube free of anticoagulant	No, additives used	[53]
5 Elnashar et al.	*PRF*	1 cycle<br>3000 rpm<br>12 minutes	No	No	Yes	"After urethroplasty, the PRF sheet was anchored over the neourethra with absorbable polyglyconate 6/0 interrupted sutures"	5-10 ml in standard plain sterile glass tube	Yes, slightly increased rpm	[45]
6 Elsayem et al.	*PRP autologous gel*	2 cycles, first "low spin centrifugation at 150×g for 5 min", second cycle "high relative centrifugal force 'g'" unknown time	Yes (tricitrate sodium)	Yes (autologous thrombin and calcium gluconate)	Not mentioned explicitly	"we applied autologous platelet gel layer after making uretheral tube (urethroplasty) and was secured using 6–0 interrupted Vicryl sutures to secure dartos flap"	8 ml for PRP production in sterile glass tube with trisodium citrate and 4ml to prepare thrombin in sterile plain tube	No, 2 cycles and additives used	[54]
7 Eryilmaz et al.	PRP graft	1 cycle<br>400×g RCF<br>10 minutes	No	Yes (calcium chlorate)	Yes	"the PRP membrane was applied between the dartos flap and the skin, and then, the skin was sutured with 5–0 resorbable sutures."	5-10 ml of whole blood, container not mentioned	No, additives used	[55]
8 Guinot et al.	*PRF membrane*	1 cycle<br>3000 rpm<br>(400×g RCF)<br>10 minutes	No	No	Yes	"This autologous PRF membrane was applied over the urethroplasty and secured using a few	5-10 ml in sterile plain glass tube	Yes, minor deviation in rpm and	[49]

							resorbable polyglyconate 7/0 interrupted sutures”		reduced time	
9	Ibrahim et al.	*PRP Layer*	1 cycle<br>3000 rpm<br>20 minutes	No	No	No	“the PRP was applied directly as a liquid/gel onto the repair site covering the neourethra. In a few minutes, PRP forms a clot-like gel layer.”	5-10 ml in sterile tube with no anticoagulants	No, applied as a liquid, not a membrane	[56]
10	Mansour et al.	*PRF membrane*	1 cycle<br>2700 rpm<br>(400×g RCF)<br>12 minutes	No	No	Yes	“the PRF membrane was secured upon urethroplasty and secured with 6/0 polyglactin few interrupted sutures”	10 ml in sterile glass tube without anticoagulant	Yes, Choukroun’s PRF – L-PRF	[46]
11	Siregar et al.	*Bio-PRF gel*	1 cycle<br>7×g RCF<br>8 minutes	No	No	Yes	“Sutured to the surrounding tissue using 6–0 polydioxanone”	10 ml in plain collection tube without anticoagulant	Yes, reduced RCF and time	[50]
12	Ural et al.	*PRF membrane*	1 cycle<br>2800 rpm<br>12 minutes	No	No	Yes	“The membrane was wrapped around the neourethra and fixed with four interrupted 8—0 polyglycolic acid sutures”	4.5 ml in citrate-free siliconized tubes	Yes	[47]
13	Wishahy et al. (fistula)	*PRF membrane*	1 cycle<br>3500 rpm<br>20 minutes	No	No	Yes	“overlying the PRF extract directly over the repair and fixation in the four corners by Vicryl 6-0 sutures”	5 ml in sterilized plastic tube with no additives	Yes, slightly increased rpm and time	[48]
